# Complex History of Organellar Introgression in *Nothofagus* Trees: Chloroplast and Mitochondrial Capture Facilitated by Natural Selection

**DOI:** 10.1002/ece3.73758

**Published:** 2026-06-05

**Authors:** Gabriela Narváez, Marie‐Laure Guillemin, Francisco Sepúlveda‐Espinoza, Daly Noll, Rodrigo A. Gutiérrez, Verónica Arana, Nicolás Bellora, Francisco A. Cubillos, Juliana A. Vianna

**Affiliations:** ^1^ Instituto de Ciencias Ambientales y Evolutivas, Facultad de Ciencias Universidad Austral de Chile Valdivia Chile; ^2^ Millennium Nucleus of Patagonian Limit of Life (LiLi) Valdivia Chile; ^3^ Millennium Institute Center for Genome Regulation (CGR) Santiago Chile; ^4^ Millennium Institute Biodiversity of Antarctic and Subantarctic Ecosystems (BASE) Santiago Chile; ^5^ Millennium Nucleus of Marine Agronomy of Seaweed Holobionts (MASH) Valdivia Chile; ^6^ Centro FONDAP de Investigación de Ecosistemas Marinos de Altas Latitudes (IDEAL) Valdivia Chile; ^7^ Departamento de Ciencias Biológicas y Biodiversidad Universidad de Los Lagos Osorno Chile; ^8^ Facultad de Ciencias Biológicas Pontificia Universidad Católica de Chile Santiago Chile; ^9^ Institute of Ecology and Biodiversity (IEB) Santiago Chile; ^10^ Millennium Institute for Integrative Biology (iBio) Santiago Chile; ^11^ Consejo Nacional de Investigaciones Científicas y Tecnológicas (CONICET) Bariloche Argentina; ^12^ Instituto de Investigaciones Forestales y Agropecuarias Bariloche (IFAB) Instituto Nacional de Tecnología Agropecuaria, Estación Experimental Bariloche – Consejo Nacional de Investigaciones Científicas y Técnicas (INTA EEA Bariloche‐CONICET) San Carlos de Bariloche Argentina; ^13^ Fundación INTECNUS, Bariloche San Carlos de Bariloche Argentina; ^14^ Departamento de Biología, Facultad de Química y Biología Universidad de Santiago de Chile Santiago Chile; ^15^ One Health Institute, Faculty of Life Sciences Universidad Andrés Bello Santiago Chile

**Keywords:** chloroplast capture, hybridization, mitochondrial capture, *Nothofagus*, organellar co‐capture, organellar introgression

## Abstract

Hybridization is widespread across diverse groups of organisms, and in some cases, organellar genomes of one species become fixed in another following hybridization and backcrossing, a phenomenon known as organelle capture. Because cytoplasmic genomes can contribute to local adaptation, organelle capture has the potential to confer a selective advantage and may be maintained by natural selection. Here, we evaluate this hypothesis in *Nothofagus* trees by analyzing chloroplast and mitochondrial genomes. Organellar phylogenies consistently separate individuals by geography rather than by species, with the two main clades mostly divided into northern and southern groups with a boundary at about 42° S, a pattern not captured by traditional nuclear markers. This major split is estimated to have originated during Pleistocene glaciations, when contraction into refugia and postglacial expansion may have been facilitated by hybridization. Chloroplast genes show signatures of positive selection in protein‐coding genes, with most signals concentrated in the northern clade. These results are consistent with potential adaptation to latitudinal and environmental gradients. In contrast, mitochondrial genes remain conserved under purifying selection, suggesting that mitochondrial patterns may be consistent with co‐introgression alongside chloroplasts. This pattern is consistent with the predominant maternal inheritance of both organelles in angiosperms, unlike gymnosperms where mitochondria are usually maternally and chloroplasts paternally inherited, a difference that may shape organelle capture dynamics. Overall, our results are consistent with a potential pattern of organelle co‐introgression involving the simultaneous introgression of chloroplast and mitochondrial genomes through hybridization. These findings provide a framework for exploring the evolutionary significance of organelle introgression and its potential role in local adaptation and speciation in trees.

## Graphical abstract

Map of sampling for each species, chloroplast and mitochondrial genome phylogeny.

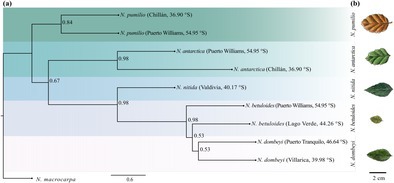

## 1 Introduction

Hybridization is a common evolutionary process across a wide range of organisms. In plants, for example, it is estimated to occur in approximately 25% of angiosperm species worldwide (Grant 1989). Recent advances in genomics and phylogenomics have substantially improved our ability to detect both recent and ancient hybridization events and to understand their genetic and evolutionary consequences (Stull et al. 2023). One such consequence is introgression, which can enhance fitness by contributing to the evolution of adaptive traits in recipient species (Abbott et al. 2013; He et al. 2019; Thawornwattana et al. 2022). As a possible outcome of hybridization, introgression leaves traceable genomic signatures reflecting these events. Through repeated backcrossing with one of the parental species, donor‐derived genomic regions may become more frequent in the recipient genome. If these regions harbor adaptive variants (i.e., are under positive selection), natural selection may promote or maintain their presence, whether in the nuclear genome or in organellar genomes such as chloroplasts or mitochondria.

Although traditionally seen as neutral or conserved, organellar genomes in plants can also play a role in these processes of hybridization and exhibit signatures of adaptive introgression. These processes are often revealed through gene tree discordance between nuclear and organellar genomes, when different genomic regions indicate conflicting evolutionary histories (Rieseberg et al. 1990; Sang and Zhong 2000; Wendel and Doyle 1998). In many taxa, this is manifested as cytonuclear discordances, where organellar phylogenies (those of chloroplasts and/or mitochondria) disagree with nuclear phylogenies. A related phenomenon, known as chloroplast or mitochondrial capture, occurs when the organellar genome of one species becomes fixed in another following hybridization and backcrossing (Rieseberg and Soltis 1991; Soltis and Kuzoff 1995). This process can produce phylogenetic patterns in which distinct species share identical or nearly identical organellar genomes, forming clades that reflect geography more than taxonomy (Lee‐Yaw et al. 2019). Such capture events are increasingly recognized as common in plants, yet their evolutionary causes and consequences remain poorly understood.

In plants, there is growing evidence for complex organelle inheritance processes, in particular, the biparental inheritance of chloroplasts and mitochondria is commonly reported (Birky 1995). Transmission modes range from strict maternal inheritance to biparental or even paternal inheritance, with about one‐third of studied species exhibiting biparental plastid inheritance (Mogensen 1996). In some cases, chloroplasts and mitochondria can be inherited from different sexes, as seen in cucumber (Kuroiwa 2010; Nagata 2010). These flexible inheritance patterns may facilitate organelle introgression and capture in hybrid zones. Several cases of chloroplast capture have been reported in angiosperms (e.g., *Betula*, Palme et al. 2004; *Quercus*, Yang, Qu et al. 2021). Nevertheless, most examples documented in flowering plants correspond to horizontal gene transfer (HGT) across genera (e.g., *Gnetum*, Won & Renner, 2003; *Geranium*, Park et al. 2015; *Amborella*, Rice et al. 2013), rather than chloroplast capture through hybridization. Unlike hybridization, which involves the fusion of gametes and subsequent backcrossing, HGT in these taxa is typically mediated by nonsexual mechanisms such as physical contact between unrelated species (e.g., through parasitism or natural grafting), often resulting in the movement of specific genomic fragments rather than entire organelle genomes. In contrast, the existence of mitochondrial capture has so far only been reported in gymnosperms such as *Abies* and *Pinus* (i.e., Semerikova et al. 2011, Willyard, Gernandt, Cooper, et al. 2021 respectively). To date, there is no evidence of mitochondrial capture in angiosperms nor of chloroplast capture in gymnosperms.

Despite the biological relevance of organelle introgression and capture in plants, few evolutionary studies have tested the importance of positive selection leading to the fixation of the same organelle parental genomes in various species sharing a common distribution range. This gap may be explained by the fact that organellar genomes encode only a small fraction of the proteins required for their function and depend heavily on nuclear genes for regulation (Millar et al. 2005). Moreover, due to their central roles in photosynthesis and cellular respiration, variation in organelle‐encoded genes has long been assumed to be efficiently removed by purifying selection (Wolfe et al. 1994). However, evidence is accumulating that cytoplasmic genomes may contribute to adaptation in plants. Cytoplasmic capture, cytoplasm‐mediated local adaptation, and even positive selection on chloroplast genes have been proposed (Budar and Roux 2011). The chloroplast, as the site of photosynthesis, plays a central role in local adaptation to light, temperature, and water availability, and several chloroplast‐encoded enzymes have been shown to evolve under positive selection (Hu et al. 2015; Gao et al. 2019; Yang, Qu, et al. 2021). Thus, replacing a native chloroplast genome with one from another species may confer a selective advantage in novel environments (Tsitrone et al. 2003; Muir and Filatov 2007; Percy et al. 2014). Mitochondria are similarly crucial for respiration and metabolic function. Although plant mitochondrial genomes are more structurally complex than their animal counterparts (Kozik et al. 2019; Li et al. 2023), coding regions are generally well conserved, and studies in *Arabidopsis*, *Carica*, and *Nicotiana* suggest strong purifying selection on mitochondrial genes (Cheng et al. 2021). The contrasting evolutionary constraints on chloroplasts and mitochondria raise intriguing questions about their respective roles in adaptation through hybridization.

*Nothofagus* (Nothofagaceae; southern beech) is a genus of Gondwanan origin, characterized by a disjunct distribution across the Southern Hemisphere, encompassing South America and Oceania (Hill 2001). The genus includes 37 extant species of trees and shrubs, along with several naturally occurring and artificial hybrids (POWO 2025), and has been traditionally subdivided into four subgenera (van Steenis 1953, 1971; Hill and Read 1991). Heenan and Smissen (2013) proposed elevating these subgenera to the rank of genera: *Nothofagus, Fuscospora, Lophozonia*, and *Trisyngyne*, but this classification has not been widely adopted outside New Zealand (Steed‐Mundin 2025; Perry et al. 2025) and Chile and Argentina still follow the traditional nomenclature of POWO (2025). Of these southern beech species, 11 occur in the southern part of South America, in Chile and Argentina (Ramírez 1987), sharing large parts of their distribution with other Nothofagaceae species (Arroyo et al. 2019). Despite a rich fossil record and well‐documented biogeographic history (Hill 2001; Heenan and Smissen 2013; Vento et al. 2022), the evolutionary relationships among South American species remain unresolved. Phylogenies based on a limited number of nuclear and chloroplast markers often conflict, exhibiting cytogenetic discordances (Peterson et al. 2010; Premoli et al. 2012), which is consistent with a history of hybridization and introgression (Acosta and Premoli 2018). This is especially evident in the subgenus *Nothofagus*, which is restricted to the Southern Hemisphere, particularly South America. Analyses of chloroplast markers and complete chloroplast genomes (Acosta and Premoli 2010; Juri et al. 2024) have revealed phylogenetic patterns in which individuals cluster by geography (e.g., north vs. south of 42° S in Argentina) rather than by species. This pattern, interpreted as chloroplast capture, suggests that historical hybridization led to organelle replacement and geographic sorting. However, previous studies have been limited in spatial scope and have not examined the potential role of natural selection in maintaining organellar variation. Furthermore, mitochondrial capture remains unexplored in the subgenus.

Here, we present the first mitochondrial genome assemblies for *Nothofagus* and reconstruct and analyze complete chloroplast and mitochondrial genomes from five species of the subgenus *Nothofagus* sampled across a broad latitudinal gradient in South America (36° S–54° S). Our goals were to evaluate whether patterns consistent with organellar capture are detectable in both genomes and assess whether natural selection may have contributed to the maintenance of organellar variation across the 42° S biogeographic divide.

In this context, we assess whether mitochondrial and chloroplast phylogenetic patterns are consistent with co‐introgression and simultaneous plastome capture. Finally, we examine local adaptation as a potential evolutionary mechanism that could contribute to the persistence of introgressed organellar genomes in *Nothofagus*.

## 2 Materials and Methods

### 2.1 Sample Collection

**FIGURE 1:**
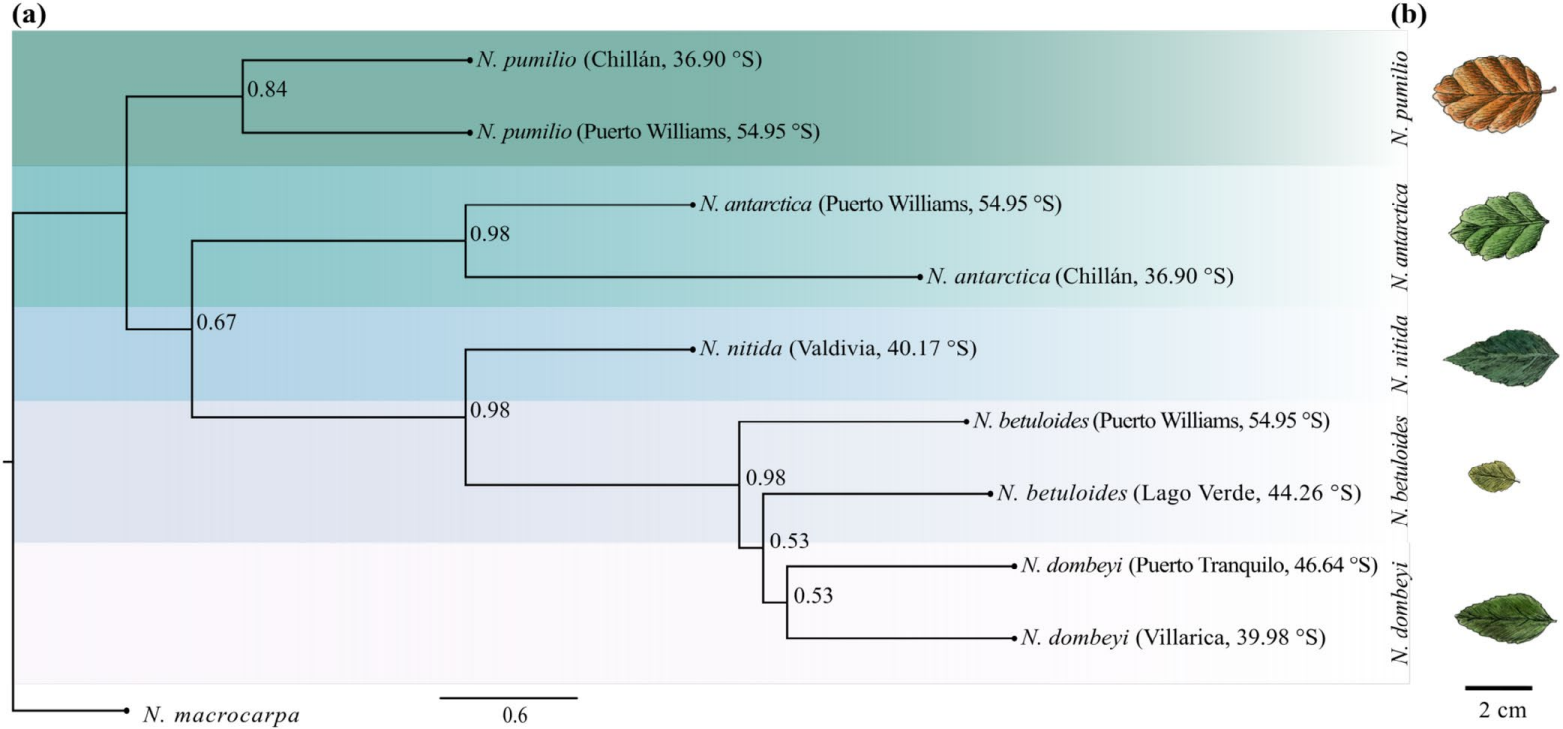
Phylogenetic tree reconstruction from nuclear data set in subgenus *Nothofagus*. (a) Species tree inferred through ASTRAL‐III. Support value is reported for each node. (b) Representative *Nothofagus* illustrated leaves.

**FIGURE 2:**
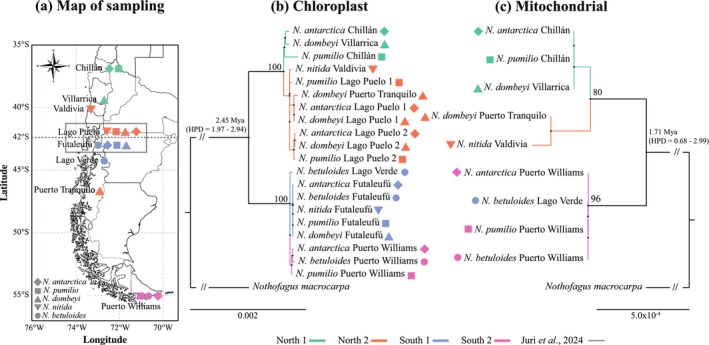
Map of sampling locations and phylogenetic relationships of the subgenus *Nothofagus* reconstructed using the Maximum Likelihood (ML) method and organelle genes. (a) Sampling locations of southern beeches used in the analysis. The main biogeographical limit of the 42° S is shown as a dotted line. (b) Phylogenetic tree based on 79 single copy CDS chloroplast sequences. The tree includes sequences of Chilean southern beeches (current study) and sequences from Juri and col. (2024), corresponding to trees sampled in Argentina in Lago Puelo and Futaleufú (inside the box in map). (c) Phylogenetic tree based on 14 single copy CDS mitochondrial sequences. In both three reconstructions, *Lophozonia macrocarpa* was used as the outgroup. Colors (maps and trees) correspond to the four main clades of chloroplast and mitochondrial sequences: North 1 (green), North 2 (orange), South 1 (lilac), and South 2 (pink). The divergence time in Mya is in bold over main split in both phylogenies (with HDP value in brackets), and the principal support value is reported for each node. Please see the Section 3 for more detail on clade compositions.

To evaluate the hypothesis of chloroplast and mitochondrial capture, we sampled the five species belonging to the subgenus *Nothofagus*: *N. antarctica* , *N. pumilio* , *N. dombeyi* , *N. betuloides*, and *N. nitida* . These samples were collected following leaf characteristics used for species delimitations (Acosta and Premoli 2010; Figure 1). However, we acknowledge the inherent taxonomic complexity of the group and the potential for misassignment due to morphological overlap and hybridization. Previous genetic studies have shown that individuals morphologically identified as *N. nitida* may cluster with *N. dombeyi* or *N. betuloides* (Mathiasen et al. 2017). To minimize this risk, all individuals included in this study were carefully evaluated to ensure consistency between morphological identification and their expected nuclear phylogenetic lineage. For *N. antarctica* , *N. pumilio* , *N. dombeyi* , and *N. betuloides*, one individual from the northernmost and one from the southernmost locality of each species' distribution in Chile were included (https://sit.conaf.cl/; Figure 2a; Table S1). For *N. nitida* , only one sample, located in Valdivia (−40.17, −73.47), was used. A single sample of *Nothofagus macrocarpa* (subgenus *Lophozonia*) was collected and used as an outgroup in phylogenetic reconstructions. Leaves from these trees were collected and preserved in silica gel until DNA extraction. Our sampling strategy was designed to maximize latitudinal coverage across the distribution of the subgenus *Nothofagus*, with emphasis on contrasting northern and southern populations. However, the limited number of individuals per species, particularly the single sample available for *N. nitida* , does not allow detailed inference regarding fine‐scale geographic structure.

### 2.2 DNA Isolation and Genome Sequencing

High‐quality genomic DNA was extracted from leaf samples using a modified cetyltrimethylammonium bromide (CTAB) protocol, as described by Sahu et al. (2012) (Data S1). The DNA samples were then sent to MedGenome (Delaware, USA) for library preparation and whole‐genome sequencing. Libraries were constructed using the TruSeq DNA Nano High Throughput Library Prep Kit (Illumina, cat. 20015965), following the manufacturer's instructions. The whole genome sequencing (~10–20 GB per sample) was performed using the 2 × 150 bp paired‐end protocol on the Illumina NovaSeq6000 platform.

### 2.3 Organelle Genome Assembly and Annotation

The chloroplast and mitochondrial genomes were assembled using GetOrganelle v1.7.7.1 (Jin et al. 2020), with *embplant_pt* and *embplant_mt* as reference databases, respectively. The assembled chloroplast and mitochondrial genome files were annotated using GeSeq (Tillich et al. 2017), applying default settings without a reference genome for the chloroplast and using *Fagus sylvatica* mitogenome (GenBank ID: NC_050960.1) as a reference genome for mitochondrial annotation. Additionally, we annotated the 11 chloroplast samples from Juri et al. (2024) to extract the coding DNA sequences (CDS) and incorporated them into this study (Table S1).

For both organelles, tRNA genes were predicted *de novo* using ARAGORN (Laslett and Canback 2004) via the CHLOROBOX platform (https://chlorobox.mpimp‐golm.mpg.de/geseq.html). Manual curation of annotation results, including the verification of initiation and termination codon positions and the boundaries of inverted repeat (IR) regions, was performed using Geneious Prime v2025.0.2 (https://www.geneious.com). Annotated chloroplast and mitochondrial genome sequences were submitted to GenBank (Table S1). Circular genome maps were generated using OGDRAW v1.3.1 (Greiner et al. 2019).

### 2.4 Structural Analysis of the Whole Organellar Genome

To analyze the length of the chloroplast and mitochondrial genomes, including the large single copy (LSC), small single copy (SSC), and IR regions of the chloroplast genome, as well as the number and function of genes in each *Nothofagus* species, we used Geneious Prime v2025.0.2 (https://www.geneious.com). For collinearity analysis of whole chloroplast and mitochondrial genomes, we employed the multiple genome alignment software Mauve v1.1.3 (Darling et al. 2004) to visualize and compare potential rearrangements.

### 2.5 Phylogenetic Reconstruction in Nothofagus

For phylogenetic reconstruction, we used three datasets: nuclear, chloroplast, and mitochondrial sequences. For the nuclear dataset, we used four genes obtained from the genomes to perform the species tree: internal transcribed spacer region (ITS), CRABS CLAW CRC gene, elongation factor 1 alpha (EF‐1a), and elongation factor 2 (EF‐2) genes. The ITS is widely and previously used in *Nothofagus* phylogenetic reconstructions (i.e., Manos 1997; Acosta and Premoli 2010; Solano et al. 2019), which can be considered a diagnostic character since it allows the identification of the different species of the subgenus *Nothofagus* (Acosta and Premoli 2010). The CRABS CLAW CRC gene is frequently employed in phylogenetics analysis in Fagaceae family (i.e., Oh and Manos 2008). Finally, the EF‐1a and EF‐2 widely used in phylogenetic reconstruction in different plants species (i.e., Fabales order Saraiva et al. 2016). All genes were extracted from whole‐genome sequencing data using BLASTn (identity threshold of 90% and default e‐value of 1e‐5).

For the organellar genomes, we extracted orthologous single‐copy coding sequences (CDS) from both chloroplast and mitochondrial genomes using Geneious Prime v2025.0.2, incorporating chloroplast CDS data from Juri et al. (2024). To ensure the reliability of the dataset used for phylogenetic inference, we performed rigorous quality control analyses, including per‐gene coverage depth assessment and read mapping inspection. Nuclear genes were analyzed individually, whereas chloroplast and mitochondrial CDSs dataset were concatenated. All datasets were aligned using MAFFT v7.4.7 (Katoh and Standley 2013) with default settings, and then filtered with BMGE v1.12 (Criscuolo and Gribaldo 2010) to remove low‐quality sequences or regions with large gaps, retaining only regions with at least 90% alignment coverage. Phylogenetic relationships were inferred using Maximum Likelihood (ML) analyses performed in IQ‐TREE (Nguyen et al. 2015) using the GTR + I + G model and 1000 bootstrap replicates for each dataset (nuclear, chloroplast, and mitochondrial). The resulting phylogenetic trees were visualized and re‐rooted for optimal representation using FigTree v1.4.4 (“https://github.com/rambaut/figtree”). Finally, for the four nuclear genes phylogenetic reconstruction, we estimated the species tree using ASTRAL‐III v.5.5 (Zhang et al. 2018). We obtained multi‐species alignments in FASTA format and performed 100 bootstrap replicates for selecting the best multi‐locus tree based on maximum likelihood support values.

### 2.6 Divergence Time Estimation

To estimate of chloroplast and mitochondrial capture events, divergence times were inferred using BEAST v2.7.7 (Bouckaert et al. 2019). Chloroplast and mitochondrial datasets were analyzed independently.

A secondary calibration was applied to the split between *Nothofagus macrocarpa* and the species of subgenus *Nothofagus* included in this study. The calibration was implemented as a lognormal prior with an offset of 73 Ma, corresponding to the minimum age inferred from *Nothofagus* pollen fossils (Dettmann et al. 1990; Swenson et al. 2001). Thus, 73 Ma was treated as a hard minimum bound, constraining the calibrated node to be no younger than this age while allowing older divergence times under the lognormal distribution. Analyses were conducted under a GTR substitution model, an uncorrelated lognormal relaxed molecular clock, and a Yule speciation prior. Three independent Markov Chain Monte Carlo (MCMC) runs were performed for 2,000,000,000 generations, sampling every 5000 steps. The extended chain length was required due to the limited number of informative sites in both organellar datasets, which resulted in slow mixing of the MCMC chains. Substitution rates were fixed according to previously published estimates: 9.6 × 10^−5^ substitutions per site per million years for the chloroplast genome (Xu et al. 2015) and 5.7 × 10^−5^ substitutions per site per million years for the mitochondrial genome (Mower et al. 2007). Convergence was assessed in Tracer v1.7 by confirming effective sample sizes (ESS) > 200 for all parameters after combining runs using LogCombiner. The first 10% of samples were discarded as burn‐in prior to generating maximum clade credibility trees with TreeAnnotator v2.7.5.

### 2.7 Genomic Signatures of Selection

To investigate the role of natural selection sustaining chloroplast and mitochondrial gene divergence and identify those under positive selection, we analyzed the 79 and 14 orthologous CDSs from the chloroplast and mitochondrial genomes, respectively, across the five species of the subgenus *Nothofagus* using a branch‐site model approach (Gharib and Robinson‐Rechavi 2013). The analysis was conducted using the principal clades of the topology of the resulting phylogeny previously established for chloroplast and mitochondrial genes (Figure 2b,c). We verified and corrected sequence length and reading frames using a three‐step process: the pre‐msa.bf script was used to correct frameshift mutations and translate the sequences into proteins. MUSCLE (Edgar 2004) was used to align amino acid sequences. The post‐msa.bf script was used to back‐translate the frameshift‐corrected nucleotide sequences. The scripts for steps 1 and 3 were obtained from https://github.com/veg/hyphy‐analyses/tree/master/codon‐msa. We investigated positive selection in chloroplast and mitochondrial protein‐coding genes by analyzing the ratio of non‐synonymous (dN) to synonymous (dS) substitution rates (ω = dN/dS). Signals of positive selection were evaluated using the mixed effects model of evolution (MEME) implemented in HyPhy https://hyphy.org/methods/selection‐methods/ (Murrell et al. 2012).

MEME employs a branch‐site framework that allows ω to vary across both sites and branches and is specifically designed to detect episodic diversifying selection acting at individual codon positions. The foreground branches tested correspond to the four main clades recovered by the organellar phylogeny: Northern clade, Clade North 1, Clade North 2 and Southern clade (Figure 2b,c). A codon position was considered a candidate under positive selection when MEME returned a likelihood ratio test (LRT) *p*‐value < 0.1. For all sites initially identified as candidates for positive selection, we applied a Benjamini–Hochberg (BH) false discovery rate (FDR) correction to the *p*‐values simultaneously. This procedure was implemented to account for multiple testing across the foreground branches and genes, ensuring a conservative and robust identification of selected sites.

## 3 Results

In this study, we successfully assembled, annotated, and characterized 12 complete organellar genomes (10 chloroplast and two mitochondrial) from *Nothofagus* trees. Chloroplast and mitochondrial sequences were obtained for nine samples of the subgenus *Nothofagus* and one sample of *N. macrocarpa*. Each sample produced hundreds of millions of short sequence reads (68,141,844 < reads < 185,798,958; 300 bp in length); between 1.51% and 6.96% of these total reads mapped to the chloroplast genome and 0.55% to 20.8% to the mitochondrial genome.

### 3.1 Organellar Structure and Comparison

The complete chloroplast genomes of southern beeches were assembled and circularized, forming a tetrameric structure with total sequence lengths ranging from 155,923 to 156,302 bp (Table S2, Figure S1a). These genomes consist of a large single‐copy (LSC) region (85,213–85,507 bp), a small single‐copy (SSC) region (18,019–18,518 bp), and a pair of IR regions (Ira and Irb) spanning 26,096–26,343 bp each. The same number of genes, exons and introns was detected in all samples studied, including *N. macrocarpa* (Table S2). The genetic composition (CDS, tRNA, rRNA) of the LSC, SSC, Ira, and Irb was also similar across all samples studied (Table S2). The chloroplast genomes encode a total of 130 genes, including duplicates: 85 protein‐coding genes (CDS) with eight exons and four introns, 37 transfer RNA (tRNA) genes, and eight ribosomal RNA (rRNA) genes (Table S3). The LSC region contained 61 CDS and 22 tRNA, while the SSC region had 11 CDS and 1 tRNA gene. The IR regions (Ira and Irb) contained six CDS, seven tRNA, and four rRNA genes each (Table S2).

The mitochondrial genome was successfully assembled and circularized for *N. dombeyi* (from Villarrica) and *N. betuloides* (from Lago Verde) (Genbank accession number: PZ404359 and PZ404358, respectively). The mitochondrial genome length was 369,560 bp for *N. dombeyi* and 441,652 bp for *N. betuloides*, forming a tetrameric structure (Figure S1b). These mitochondrial genomes encode a total of 47 genes, including 32 CDS, 12 tRNA, and three rRNA genes (Table S4).

A comparative chloroplast co‐linearity analysis among the five *Nothofagus* species revealed no differences in gene arrangements within chloroplast syntenic blocks (Figure S2), suggesting a conserved structure. Due to lack of samples with complete mitochondrial assembly, a synteny analysis for the mitochondrial genome could not be conducted.

### 3.2 Phylogenetic Analyses

We reconstructed individual Maximum Likelihood (ML) phylogenies for the four nuclear genes analyzed: ITS (617 bp), CRC (1118 bp), EF‐1α (1777 bp), and EF‐2 (2450 bp). All gene trees exhibited a congruent topology for the *Nothofagus* subgenus (Figure S3), consistent with previous studies (i.e., Manos 1997; Acosta and Premoli 2010; Solano et al. 2019). Similarly, the species tree generated by ASTRAL‐III (Figure 1), using the individual ML gene trees as input, resulted in a topology where individuals of the same species grouped together in the same clade. Within this topology, the deciduous species *N. pumilio* (BS = 0.84) is ancestral to all other *Nothofagus* species, followed by a sister clade of *N. antarctica* (BS = 0.98). Additionally, the evergreen species (*N. nitida, N. betuloides*, and *N. dombeyi* ) formed a supported clade (BS = 0.98). In this group, *N. nitida* is ancestral to the others, while *N. betuloides* forms a paraphyletic group, in contrast to the monophyletic *N. dombeyi*.

For the chloroplast, we used 79 orthologous single‐copy CDS, corresponding to a concatenated sequence of 67,300 bp for tree reconstruction, with a percentage of fold‐coverage per sample between 1316× and 12,199× (Table S5). The Maximum Likelihood (ML) phylogenetic analysis identified two major supported clusters (BS = 100), separating *Nothofagus* species into a northern and southern clade (Figure 2b). The northern clade included all samples located north of the 42° S biogeographic limit regardless of species, as well as one *N. dombeyi* sample from Puerto Tranquilo (46.64° S). The southern clade includes the remaining samples located south of 42° S. The northern clade was further divided into two well‐supported sub‐clades (BS = 100): North 1 (including one *N. antarctica* and one *N. pumilio* from Chillán [36.90° S], and one *N. dombeyi* from Villarrica [39.38° S]); and North 2 (sampled from Valdivia (40° S) to Lago Puelo (42.21° S), on both sides of the Andes, plus *N. dombeyi* from Puerto Tranquilo). Similarly, the southern clade was subdivided into two sub‐clades (BS = 100): South 1 (from Lago Puelo [42.11° S] to Lago Verde [44.2° S]) and South 2 (samples from Puerto Williams [54.95° S]). Divergence time estimates revealed that the split between northern and southern chloroplast clades occurred approximately 2.45 million years ago (Mya), during the Pleistocene (highest posterior densities (HPD = 1.97–2.94)). For the internal nodes of the trees, within the northern and southern clades (North 1—North 2 and South 1—South 2), were associated with lower phylogenetic resolution and broad 95% HPD intervals, reflecting limited signal for dating these more recent divergences. Accordingly, divergence times for these internal splits should be interpreted with caution and not shown in this work.

**FIGURE 3:**
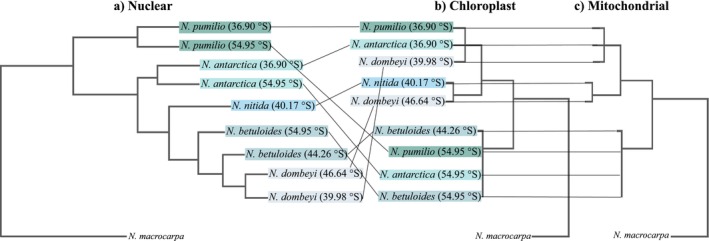
Schematic representation of discordance cytonuclear in subgenus *Nothofagus*. (a) Concatenated nuclear gene tree from ASTRAL‐III. (b) Orthologues CDS of chloroplast genome. (c) Orthologues CDS of mitochondrial genome.

For the mitochondrial genome, we used 13 orthologous single‐copy CDS, corresponding to a concatenated sequence of 9800 bp, with a percentage of fold‐coverage per sample between 189× and 1553× (Tables S5 and S6). The ML mitochondrial phylogenetic tree is congruent with the topology obtained using chloroplast data, while both differ from the species tree based on nuclear genes (Figure 3), revealing two moderately supported clades (BS = 100), separating all trees sampled north of the 42° S and *N. dombeyi* from Puerto Tranquilo, from all remaining samples collected south of the 42° S (Figure 2c). Within the northern clade, trees from Chillán and Villarica cluster together and the sister clade (BS = 80) was composed of *N. nitida* (Valdivia) and *N. dombeyi* (Puerto Tranquilo). No clear substructure was observed within the southern clade: *N. betuloides* (from Lago Verde) and *N. antarctica* , *N. pumilio* and *N. betuloides* from Puerto Williams, all belonged to this clade. Divergence time estimated using mitochondrial CDS sequences, placed the split between northern and southern clades at approximately 1.71 Mya during the Pleistocene (95% HPD = 0.68–2.99 Mya). Estimates for more recent nodes were associated with wider HPD intervals (similarly to the chloroplast phylogenetic reconstruction), reflecting the limited phylogenetic signal available for resolving recent divergences in this dataset.

### 3.3 Variation in Gene Length and Number in Chloroplast Genomes

We observed differences in gene length in several chloroplast genes, mostly between N. *macrocarpa* and the samples from the *Nothofagus* subgenus (e.g., *acc*D, *inf*A, *ndh*F, *ndh*K, *psb*T, *rps*3, *rps*16, *rps*18, *ccs*A, *ndh*I, *rpl*22—large copy, *rpo*A—large copy, *ycf*1—large copy, and *ycf*2—both short and large copies; Table S7). The SSC region was identified as a hotspot for length variation. Additionally, *rpl*22, *rpo*A, *ycf*1 (large copy), and *ycf*2 (both short and large copies) also displayed length differences between the northern and southern clades. One 6‐bp insertion was observed in *ccs*A in *N. nitida* (Valdivia) compared to all other samples. Finally, *ndh*I exhibited a more complex variation pattern with five different CDS lengths ranging from 510 to 534 bp (Table S6). No variation in gene length was detected among the 14 orthologous mitochondrial genes analyzed.

*Nothofagus macrocarpa* had an IR length of 26,096 bp (Table S2). Within the *Nothofagus* subgenus, the longest IR (26,343 bp) was found in *N. nitida* (Valdivia), while the shortest IR (26,272 bp) occurred in southern clade samples ( *N. antarctica* , *N. pumilio* from Puerto Williams and *N. betuloides* from Puerto Williams and Lago Verde). In most cases, Ira and Irb were equal in length, but in *N. dombeyi* (Puerto Tranquilo) and *N. nitida* (Valdivia), Ira was longer than Irb (Table S2).

### 3.4 Signature of Selection

Synonymous substitutions (dS) accumulate nearly neutrally, whereas nonsynonymous substitutions (dN) are subjected to selective pressures. A dN/dS ratio (ω) > 1.0 indicates positive selection, while values ranging between 0.5 and 1.0 suggest relaxed selection (Murrell et al. 2012). We identified several genes with signatures consistent with episodic positive selection, supported by log‐likelihood values and likelihood ratio tests (LRT) (Table 1). The branch‐site model detected significant positive selection genes, supported by Likelihood Ratio Test (LRT) and Benjamini–Hochberg FDR corrected *q*‐values (all < 0.10, Table 1). MEME detected candidate sites under episodic diversifying selection in chloroplast genes of the northern clade (*matK, ndhC, ndhF, rps16*, and *ycf2* at one codon position, and *rpoC2* and *ycf1* at three codon positions) and in southern clade (*psbC* at one codon position). Additional candidate sites were identified in North1 (*ndhF, ndhH*, and *rpoC2*, one codon each) and North2 (*matK, rpoB* at one codon position, and *ycf1* at two codon positions). No signatures of positive selection were detected in mitochondrial genes. These results should be interpreted as identifying candidate genes for future functional validation rather than as direct mechanistic proof of adaptive fixation.

**TABLE 1:** Likelihood ratio test (LRT) results for candidates sites under episodic positive selection in *Nothofagus* chloroplast genes. Results are based on the mixed effects, model of evolution (MEME) implemented in HyPhy cross four foreground branches. A codon position was considered a candidate under positive selection at Likelihood Ratio Test (LRT) *p*‐value < 0.1. To account for multiple testing, *q*‐values were calculated using Benjamini–Hochberg (BH) false discovery rate (FDR) correction applied simultaneously to all 19 identified candidate sites. All reported sites remained significant at *q* < 0.10. The null model assumes no positive selection (*d*_N_ ≤ *d*_S_), whereas the alternative model allows for a proportion of branches to evolve under positive selection (*d*_N_ > *d*_S_).

Foreground branch	Gene	Gene length (bp)	Codon position	Mutation	Protein code	LRT	*p*	*q*
Northern clade	*matK*	1524	504	aCc>aGc	Thr>Ser	3.257	0.0933	0.0962
	*ndhC*	363	26	Cta>Ata	Leu>Ile	4.628	0.0457	0.0875
	*ndhF*	1458	453	aCc>aTc	Thr>Ile	6.261	0.0197	0.0875
	*rpoC2*	4167	875	gTg>gCg	Val>Ala	3.551	0.0800	0.0962
	*rpoC2*	4167	1006	tCg>tTg	Ser>Leu	4.844	0.0409	0.0875
	*rpoC2*	4167	1265	aTc>aCc	Ile>Thr	3.922	0.0659	0.0894
	*rps16*	255	82	Ctt>Ttt	Leu>Phe	4.451	0.0501	0.0875
	*ycf1*	1239	523	Tac>Gac	Tyr>Asp	4.612	0.0461	0.0875
	*ycf1*	1239	560	Att>Ttt	Ile>Phe	4.107	0.0599	0.0875
	*ycf1*	1239	984	cAg>cGg	Gln>Arg	4.105	0.0599	0.0875
	*ycf2*	6816–6822^a^	786	ttG>ttT	Leu>Phe	3.198	0.0962	0.0962
Clade North 1	*ndhF*	1458	453	aCc>aTc	Thr>Iso	6.257	0.0198	0.0875
	*ndhH*	1182	299	Agg>Ggg	Arg>Gly	4.165	0.0581	0.0875
	*rpoC2*	4167	746	gCa>gAa	Ala>Glu	4.916	0.0394	0.0875
Clade North 2	*matK*	1524	222	Ttc>Ctc	Phe>Leu	5.290	0.0325	0.0875
	*rpoB*	3219	303	cTt>cGt	Leu>Arg	3.528	0.0810	0.0962
	*ycf1*	1239	523	Tac>Gac	Tyr>Asp	5.948	0.0232	0.0875
	*ycf1*	1239	560	Att>Ttt	Ile>Phe	4.578	0.0469	0.0875
Southern clade	*psbC*	1416	370	Ccc>Tcc	Pro>Ser	3.351	0.0888	0.0962

^a^ Variation length (Table S5).

## 4 Discussion

Our study provides evidence consistent with introgression having profoundly shaped the evolutionary history of the *Nothofagus* in South America. Phylogenetic trees obtained for CDS organelle genes differ markedly from the nuclear phylogeny based on four genes and from morphological data, splitting samples by geography rather than by species. Both chloroplast and mitochondrial genomes consistently recover two main lineages, broadly corresponding to samples collected north and south of the 42° S biogeographic boundary. These genomes, especially the plastome, were able to detect geographical clustering, separating individuals inside the northern and southern clades. Molecular dating suggests that the two main lineages diverged during the Pleistocene glaciations. We propose that the observed patterns are consistent with the joint introgression of both organelles by various *Nothofagus* species when their distribution contracted to distinct glacial refugia, and that this phenomenon may reflect multiple introgression events, explaining the low genetic diversity, short branch lengths, and poor resolution found in both organelle genomes within each geographic subclade. However, additional sampling would further help clarify the recurrence of the introgression events. Our results reveal signatures consistent with episodic positive selection in chloroplast genes, as detected by MEME dN/dS analyses (Murrell et al. 2012), suggesting that adaptive evolution may have played a role in shaping chloroplast genome variation across clades. Our results are consistent with the hypothesis that natural selection may have contributed to the retention of introgressed chloroplast genomes, whereas stronger purifying selection may impose greater constraints on mitochondrial evolution. Notably, our work provides the first mitochondrial genome assemblies for the family Nothofagaceae, addressing a genomic resources gap for this southern hemisphere tree lineage. In addition, our findings are consistent with a potential case of organelle co‐capture in plants (proposed name), involving the simultaneous capture of chloroplast and mitochondrial genomes through hybridization and introgression (representative scheme in Figure S4). Rather than representing a rare anomaly, organelle genome capture may constitute an evolutionary process that contributes to local adaptation and diversification, with implications for evolutionary biology, conservation, and systematics.

A key finding of this study is the discovery of organelle genome co‐capture in the subgenus *Nothofagus*. While plastid capture had already been described in southern beeches (Acosta and Premoli 2010; Juri et al. 2024), our study provides the first genome‐wide evidence of chloroplast capture across the full geographic range of the subgenus. These results align with findings in other angiosperm lineages, where historical hybridization and introgression leading to chloroplast capture has been reported in trees such as *Betula* and *Quercus* (Fagales; Palme et al. 2004; Yang, Qu, et al. 2021; Yang, Zhou, et al. 2021), *Eucalyptus* (Nevill et al. 2014), and in other taxa like the Rosaceae (Liu et al. 2020). However, in these groups, the potential for mitochondrial co‐capture remains unknown, as no studies to date have jointly analyzed chloroplast and mitochondrial genomes. Indeed, mitochondrial capture has been much less frequently reported in plants than in animals, largely due to the structural complexity of plant mitogenomes, including their large size variation, abundance of repetitive elements, which generate considerable challenges for accurate assembly and annotation (Kozik et al. 2019). This complexity is particularly evident in the order Fagales, where mitogenomes can exist as multiple circular or linear chromosomes (Feng et al. 2021). For instance, within the Betulaceae, *Carpinus cordata* possesses three circular mitochondrial chromosomes, while *Alnus glutinosa* exhibits up to 16 linear fragments (Feng et al. 2021). Such structural fluidness, driven by frequent recombination and rearrangements (Sloan et al. 2012; Gualberto and Newton 2017), poses significant risks for whole‐genome alignments, which can be prone to artifacts due to nonhomologous regions. Here we report a complete circular assembly of mitogenomes for two *Nothofagus* species (*N. betuloides* and *N. dombeyi* ). These represent the first mitochondrial genomes not only for the genus, but also for the entire Nothofagaceae family. *Nothofagus* mitogenomes fall within the expected size range for the Fagales order (from 388,038 bp in *Castanea mollissima* to 922,154 bp in *Carpinus cordata*; Feng et al. 2021; *N. dombeyi* 369,560 bp and *N. betuloides* 441,652 bp) with the number of CDS also similar to the range reported for the order (from 34 to 38 CDS; *N. dombeyi* and *N. betuloides* 32 CDS). To date, reports of mitochondrial capture in plants have been limited to gymnosperms, specifically, it has been reported in the genera *Abies* (Semerikova et al. 2011, 2018; Semerikova and Semerikov 2014a, 2014b; Shao and Xiang 2015; Uchiyama et al. 2023) and *Pinus* (Willyard, Gernandt, Cooper, et al. 2021; Willyard, Gernandt, López‐Reyes, and Potter 2021). In both *Abies* (studies based on three mitochondrial DNA sequences; Semerikova and Semerikov 2014b) and *Pinus* (studies based on survey of repeated motifs in nad1 intron 2, Willyard, Gernandt, López‐Reyes, and Potter 2021) mitochondrial markers cluster samples by geography instead of by species (mitochondrial capture). Contrastingly, plastid phylogenies classically group samples by species and chloroplast phylogenies are largely congruent with nuclear genes and morphological traits in these two genera (Semerikova and Semerikov 2014a; Shao and Xiang 2015). These contrasting patterns, with the two organelles showing widely distinct phylogenetic reconstructions have been associated with a process of mitochondrial capture without accompanying plastid capture in both *Abies* and *Pinu*s. In fact, for *Abies*, it has been proposed that the chloroplast undergoes recombination during the capture process (Semerikova and Semerikov 2014a), probably due to the formation of hybrid heteroplasmic individuals (Leducq et al. 2017). The branching pattern of the mitochondrial phylogenetic tree closely follows that observed for the chloroplast, supporting the idea of organelle co‐capture in our study. Unlike gymnosperms, where most species exhibit maternal inheritance of mitochondrial DNA but paternal inheritance of chloroplast DNA, in angiosperms, maternal inheritance of both organelles is predominant (Birky 2008; Greiner et al. 2015). We hypothesize that this maternal inheritance of both chloroplasts and mitochondria facilitates the organelle co‐capture phenomenon observed in *Nothofagus*. In *Nothofagus*, only chloroplast inheritance has been empirically studied so far, with evidence supporting its maternal transmission (Premoli et al. 2012). However, in a related species such as oak ( *Quercus robur* , Fagaceae), through intraspecific crosses, both chloroplast and mitochondrial genomes were shown to be maternally inherited (Dumolin et al. 1995). We propose that in this subgenus, hybridization events followed by repeated backcrossing to the paternal species result in the stable co‐introgression of both chloroplasts and mitochondria. In plant lineages characterized by high levels of hybridization and recurrent backcrossing, chloroplast capture has been frequently reported (i.e., some *Quercus*, Leroy et al. 2017, Zhou et al. 2024, and other Fagales, Yang, Zhou, et al. 2021). Several studies, based on cytogenetic (Acosta and Premoli 2018) or morphological and molecular tools, have reported support for hybridization between *Nothofagus* species. Hybrids have been reported between *N. dombeyi* and *N. betuloides* (Donoso and Atienza 1983), *N. antarctica* and *N. pumilio* (Quiroga et al. 2005) and *N. dombeyi* and *N. antarctica* (Rozzi 2024 pers. comm.), reinforcing the idea that incomplete reproductive barriers in this subgenus allow for successful hybrid formation and organelle transfer.

The timing of divergence between the two main clades, as recovered from chloroplast and mitochondrial data, is consistent, further supporting a shared evolutionary history. The chloroplast split between the northern and southern clades was dated to ~2.45 Mya (HPD: 1.97–2.94), and the mitochondrial divergence to ~1.71 Mya (HPD: 0.68–2.99). However, the mitochondrial phylogeny was reconstructed from only 13 orthologous single‐copy CDS genes, resulting in broader uncertainty around divergence‐time estimates and reduced phylogenetic resolution for more recent splits. Nevertheless, both estimates fall within the Pleistocene, a period marked by repeated glaciations and episodes of population contraction and expansion likely promoting lineage isolation as well as opportunities for secondary contact and hybridization (Villagrán 2018; Rivera et al. 2024). The discrepancy with previous Miocene estimates based on chloroplast intergenic regions (Premoli et al. 2012) likely reflects differences in mutation rates between coding and noncoding regions, as well as saturation of substitutions in rapidly evolving intergenic sequences (Christensen 2014). In addition to this main split, we observe other recent internal splits in both northern and southern regions, resulting in four geographical areas (mainly visible in chloroplasts) along the latitudinal distribution of *Nothofagus*. Repeated introgression during glacial periods may have progressively replaced earlier organellar variation, giving rise to the distinct lineages observed today. We hypothesize that the observed latitudinal structuring of organellar genomes reflects the location of Pleistocene glacial refugia (especially the ones formed during the upper Pleistocene), with subsequent postglacial bidirectional expansions shaping the current distribution of chloroplast and mitochondrial lineages. Various potential glacial refugia have been proposed specifically for *Nothofagus* with some located in the Andes (i.e., Quilanlahue around 40° S, Soliani et al. 2012), while others have been proposed as far south as Tierra del Fuego (around 54° S, Premoli et al. 2010; Soliani et al. 2012). Indeed, Sersic et al. (2011) propose eleven major areas where plants may have persisted in situ in South America, like Chilean Coastal range north of 42° S and Chiloé island, longitudinal zone located in the eastern flanks of the Andes from 38° to 42° S, scattered areas located in the high‐Andean region north of 38° S, San Jorge Gulf (approximately 46° to 47° S), Tierra del Fuego and others. These refugia could have acted as sources of migrants during the recolonization following the ice retreat, with northern lineages expanding southward and southern lineages northward, both converging around 42° S—a well‐documented biogeographic transition zone. Similar dynamics of population contraction in glacial refugia followed by rapid recolonization have also been previously reported for other tree species in Patagonia (e.g., *Fitzroya cupressoides*, Premoli et al. 2000; *Austrocedrus chilensis* , Pastorino and Gallo 2002). Periods of population contraction and expansion could both be favorable to organelle capture. Hybridization and organelle introgression are more likely in small, overlapping populations (as glacial refugia), where repeated contact increases the probability of interspecific crosses (Hewitt 2000). Strong genetic drift during rapid postglacial recolonization events can further facilitate the fixation of introgressed organelles (Petit et al. 2003). In Abies, it has been proposed that the mitochondrial capture occurred during the genus expansion into Eurasia from North America (Semerikova et al. 2018). In *Nothofagus*, new extensive sampling in glacial refugia along the recolonization roads may understand fine processes occurring during periods of contraction and expansion.

Interestingly, the effect of selection in shaping the evolutionary trajectories of chloroplasts and mitochondria appears as distinctive, with various chloroplast genes displaying signatures of adaptive evolution while all mitochondrial genes seem constrained by purifying selection. Within the Northern clade (36°–42° S), signals of positive selection in chloroplast genes are not uniformly distributed but instead show a clear latitudinal structure. In the northernmost range (36°–40° S), where the Mediterranean‐type conditions dominate with prolonged summer droughts, high solar radiation, and strong seasonality in temperature and photoperiod (Armesto et al. 2007; Deitch et al. 2017), signs of positive selection were detected in the genes *ndh*F, *ndh*H, and *rpo*C2. These genes are directly involved in the regulation of photosynthesis (*ndh* genes, Martín and Sabater 2010) and transcription (*rpo* genes, Serino and Maliga 1998), functions that may be crucial for coping with water stress, intense radiation, and fluctuating energy demands. In Fagales, the *rpoC*2 gene has been previously reported under positive selection in *Quercus* (Liu et al. 2021). The signature of selection in the *rpoC*2 gene has been associated with adaptation to sunny environments in wild rice (*Oryza australensis*; Gao et al. 2019) and increased water use efficiency in the common bean ( *Phaseolus vulgaris* ; Ruiz‐Nieto et al. 2015). In contrast, in the transitional zone further south (40°–42° S), where temperate rainy climates begin to prevail and cloud cover, humidity, and lower temperature variability define local environments (Muñoz et al. 2016; Orrego‐Verdugo et al. 2021), a different set of genes—*mat*K, *rpo*B, and *ycf*1—were found under positive selection. These genes are associated with transcription (*mat*K, Hao et al. 2010 and *rpo* genes, Serino and Maliga 1998) and synthesis of inner membrane complex involved in protein translocation (Kikuchi et al. 2013), also associated to synthesis of Tic214, a vital component in plants (*ycf*1 gene, Dong et al. 2015). In the Fagales *Morella rubra* , *Juglans regia* , *Castanea mollissima* and *Ostrya rehderiana*, signatures of positive selection have also been detected in the *rpo* and *ycf*1 genes, with the *ycf*1 exhibiting the highest K_A_ (nonsynonymous substitutions) value (Liu et al. 2017). *ycf*1 is a target of positive selection in diverse groups of plants (e.g., *Pinus*, Parks et al. 2009; Caryophyllaceae, Sloan et al. 2014, Styracaceae, Zheng et al. 2025), a pattern related to adaptability of plants under adverse conditions, strengthening their survival and reproductive capabilities (Zheng et al. 2025). In contrast, species of the Southern clade, distributed between 42° S and extending as far south as Cape Horn (56° S), inhabit markedly different environmental conditions. This austral region is characterized by acidic, phosphorus‐poor soils, frequent winter precipitation—especially in channels and fjords—and consistently strong winds near water bodies, and temperatures ranging from 5°C in winter to 20°C in summer (Schneider et al. 2003). In Patagonia, *Nothofagus* typically grow in shaded forests, scrublands, or mountain slopes where direct sunlight is limited by dense canopy cover (Toro‐Manríquez et al. 2020). The low light availability in these habitats could exert distinct selective pressures on photosynthetic efficiency. Supporting this, the gene *psb*C—found under positive selection in the Southern clade—is known to play a key role in photosystem II by binding chlorophyll and facilitating light‐induced photochemical reactions (Rochaix et al. 1989; Cai et al. 2010). Interestingly, this gene has also been identified under positive selection in *Populus tremula* , a species native to the cool temperate regions of Europe and Asia (Huang et al. 2017), which reinforces its potential role in adaptation to low‐light, cold environments. Together, these results are consistent with the possibility that chloroplast genomes may contribute to local adaptation under different climatic regimes. We propose that the fixation of geographically structured chloroplast genomes may have been facilitated by positive selection acting on key functional genes, potentially contributing to adaptation across the ecological gradients that characterize southern South America. In contrast to the chloroplast, the mitochondrial genome of *Nothofagus* exhibits strong signatures of purifying selection. This pattern is consistent with findings reported for most land plants (except for a few examples, such as some Lamiales: Li et al. 2021); where purifying selection has been suggested to act more strongly on mitochondrial than chloroplast genes (Sloan 2013). Mitochondrial genes are functionally constrained, and their conservation is likely to limit the accumulation of substitutions, even after introgression. This contrast between candidate signals of positive selection in chloroplasts and strong purifying selection in mitochondria suggests different evolutionary dynamics between the two organellar genomes. We propose that the retention of introgressed mitochondrial lineages in *Nothofagus* may have occurred largely as a byproduct of co‐introgression with chloroplast genomes, with mitochondria potentially being maintained through hitchhiking during hybridization and backcrossing rather than through direct adaptive advantage. However, our analyses identify candidate signals of selection and do not establish a direct relationship between organellar haplotypes and environmental variables. Future studies incorporating explicit genotype–environment association analyses will be necessary to test these hypothesis.

Our findings have important implications for understanding how hybridization and selection may contribute to shaping the evolutionary trajectory of *Nothofagus* species. These trees exhibit a dynamic history of lineage divergence, secondary contact, and introgression, with our results suggesting that selection may have contributed to patterns of organellar genome composition, particularly in the chloroplast. As genomic resources expand in this group, together with increasing resolution provided by long‐read sequencing, future research will be able to evaluate whether organelle co‐capture represents a recurrent phenomenon in *Nothofagus* and other angiosperms, contrasting with the pattern of mitochondrial capture without accompanied chloroplast introgression that has been described in gymnosperms. Future studies incorporating denser geographic sampling across both allopatric and sympatric populations, particularly around the 42° S boundary and putative contact zones, will be essential to better resolve fine‐scale spatial structure, identify ongoing or historical introgression, and clarify the temporal dynamics and potential recurrence of organelle introgression in *Nothofagus*. Such advances will help determine whether co‐capture reflects a broader evolutionary process in hybridizing angiosperm lineages or a more lineage‐specific outcome only detected in *Nothofagus*. Moreover, this work highlights that classic chloroplast markers (e.g., *matK*, *rbcL*) and mitochondrial genes (e.g., *cox*1) may yield misleading phylogenetic inferences in groups where introgression and organelle capture obscure species relationships. Future phylogenetic and population genomic studies in plants should therefore move beyond organellar genes, incorporating nuclear data through whole‐genome sequencing or exon‐capture approaches. This shift will not only improve species delimitation in complex plant groups but also establish new paradigms for studying reticulate evolution and the role of organelles in shaping adaptive potential. These insights will be valuable for conservation, as recognizing hybrid zones and introgressed lineages is essential for identifying evolutionary significant units (ESUs) and for predicting the adaptive capacity of these forests under rapid climate change.

## Author Contributions

**Gabriela Narváez:** conceptualization (equal), data curation (equal), formal analysis (equal), investigation (equal), methodology (equal), writing – original draft (lead), writing – review and editing (equal). **Marie‐Laure Guillemin:** formal analysis (equal), investigation (equal), methodology (equal), project administration (equal), writing – original draft (equal), writing – review and editing (equal). **Francisco Sepúlveda‐Espinoza:** data curation (equal), investigation (equal), writing – review and editing (equal). **Daly Noll:** data curation (equal), formal analysis (equal), investigation (equal), methodology (equal), writing – review and editing (equal). **Rodrigo A. Gutiérrez:** conceptualization (equal), data curation (equal), investigation (equal), writing – review and editing (equal). **Verónica Arana:** conceptualization (equal), data curation (equal), formal analysis (equal), funding acquisition (equal), investigation (equal), methodology (equal), project administration (equal), writing – original draft (equal), writing – review and editing (equal). **Nicolás Bellora:** conceptualization (equal), formal analysis (equal), investigation (equal), methodology (equal), writing – review and editing (equal). **Francisco A. Cubillos:** funding acquisition (equal), writing – review and editing (equal). **Juliana A. Vianna:** conceptualization (lead), data curation (equal), formal analysis (equal), funding acquisition (lead), investigation (lead), methodology (lead), project administration (lead), writing – original draft (lead).

## Funding

We are grateful for the financial support provided by ANID subdirección de capital humano, doctorado nacional 2022‐21231780 and the following Institutions ANID—Programa Iniciativa Milenio—NCN2021_050—LILI—ICN2021_044—CGR—ICN2021_002—BASE, iBIO ICN17_022, and ANID BASAL IEB FB210006.

## Conflicts of Interest

The authors declare no conflicts of interest.

## Supporting information


**Figure S1:** Representative chloroplast (left) and mitochondrial (right) genome maps of *Nothofagus* spp. Genes located inside the circle are transcribed clockwise, and genes outside are transcribed counterclockwise. The light gray inner circle corresponds to the AT content and the dark gray to the GC content. Genes belonging to different functional groups are shown in different colors. For the chloroplast genome, the position of the LSC region, SSC region, and two IRs (IRa and IRb) are also indicated. In the figure, the chloroplast genome corresponds to *Nothofagus antarctica* Chillán and the mitochondrial genome belongs to *Nothofagus betuloides* Lago Verde.
**Figure S2:** Comparison of the complete chloroplast genome of all datasets to *Nothofagu*s (samples are described in Table S1). Two independent Mauve analysis are presented including (a) all samples of the Northern clade and (b) all samples of the Southern clade. More details on sample clustering within the Northern and Southern clade are given in Figure 2b. Local collinear blocks are represented by blocks of the same color connected by vertical lines. Horizontal short gray lines represent IRa and IRb regions respectively.
**Figure S3:** Phylogenetic tree reconstruction based on nuclear genes. Maximum Likelihood (ML) tree inferred from ITS, CRC, EF‐1a and EF‐2 genes, respectively.
**Figure S4:** Schematic representation of organellar co‐capture. Maternal organelles are transferred and captured during hybridization and successive backcrossing events in this schematic representation.
**Table S1:** Samples coordinates of *Nothofagus* spp. and GenBank accession number.
**Table S2:** Total sequence length and structural characterization of chloroplast genomes of six *Nothofagus* species from this study. Samples are ordered following the tree presented in Figure 2b.
**Table S3:** Gene composition of chloroplast genomes of six species of *Nothofagus* spp.
**Table S4:** Gene composition of mitochondrial genomes of two samples of *Nothofagus*, one of *N. dombeyi* (Villarrica) and one of *N. betuloides* (Lago Verde), for which assembled and circularized sequences were obtained.
**Table S5:** Summary of read mapping statistics and sequencing depth for 79 chloroplast protein‐coding genes across *Nothofagus* species. Values represent the number of mapped reads, with average fold‐coverage (X) indicated in parentheses obtained thought raw reads.
**Table S6:** Summary of read mapping statistics and sequencing depth for 13 mitochondrial protein‐coding genes across *Nothofagus* species. Values represent the number of mapped reads, with average fold‐coverage (X) indicated in parentheses obtained thought raw reads.
**Table S7:** Variation in chloroplast gene length among *Nothofagus* spp.

## Data Availability

Nuclear regions, mitochondrial CDS genes, and chloroplast and mitochondrial annotations are deposited to NCBI (details in Table S1).
