# Supplementary material for: Complex History of Organellar Introgression in Nothofagus Trees: Chloroplast and Mitochondrial Capture Facilitated by Natural Selection
Source: Ecol Evol. 2026 Jun 5;16(6):e73758. doi: 10.1002/ece3.73758 (PMC13241583; doi:10.1002/ece3.73758)
Supplement: Supplementary file 1 — Figure S1: Representative chloroplast (left) and mitochondrial (right) genome maps of Nothofagus spp. Genes located inside the circle are transcribed clockwise, and genes outside are transcribed counterclockwise. The light gray inner circle corresponds to the AT content and the dark gray to the GC content. Genes belonging to different functional groups are shown in different colors. For the chloroplast genome, the position of the LSC region, SSC region, and two IRs (IRa and IRb) are also indicated. In the figure, the chloroplast genome corresponds to Nothofagus antarctica Chillán and the mitochondrial genome belongs to Nothofagus betuloides Lago Verde. Figure S2: Comparison of the complete chloroplast genome of all datasets to Nothofagus (samples are described in Table S1). Two independent Mauve analysis are presented including (a) all samples of the Northern clade and (b) all samples of the Southern clade. More details on sample clustering within the Northern and Southern clade are given in Figure 2b. Local collinear blocks are represented by blocks of the same color connected by vertical lines. Horizontal short gray lines represent IRa and IRb regions respectively. Figure S3: Phylogenetic tree reconstruction based on nuclear genes. Maximum Likelihood (ML) tree inferred from ITS, CRC, EF‐1a and EF‐2 genes, respectively. Figure S4: Schematic representation of organellar co‐capture. Maternal organelles are transferred and captured during hybridization and successive backcrossing events in this schematic representation. Table S1: Samples coordinates of Nothofagus spp. and GenBank accession number. Table S2: Total sequence length and structural characterization of chloroplast genomes of six Nothofagus species from this study. Samples are ordered following the tree presented in Figure 2b. Table S3: Gene composition of chloroplast genomes of six species of Nothofagus spp. Table S4: Gene composition of mitochondrial genomes of two samples of Nothofagus, one of N. dombeyi [file ECE3-16-e73758-s001.docx]

**Supplementary material.**

**DNA extraction protocol modified for *Nothofagus* species collected in silica gel.**

Modified from Sahu and col. (2012) by Gabriela Narváez.

**NOTE:** Small aliquot of suspension and extraction buffer should be prepared and add B-mEtOH just before use. Any unused buffer should be discarded. Due to the use of this reagent, all procedures should be performed under a fume hood.

1. Weigh 10–12 mg of dehydrated plant tissue (stored in silica gel) in grinding tubes with a tungsten bead.
2. Grind in a homogenizer for 10 s at 1500 RPM in dry conditions, then carefully remove the bead and add a spatula tip of PVP.
3. Add 500 µl of preheated suspension buffer (60 °C) containing 0.2% B-mEtOH and vortex immediately (if tissue sticks to the bottom, remove it with a tip).
4. Incubate at 60 °C for 40 min at 1200 RPM (in a thermoshaker).
5. Centrifuge at 12,000 RPM for 10 min at room temperature (RT).
6. Transfer 450 µl of the supernatant to a new tube, add 1 ml of preheated extraction buffer (60 °C) containing 1% B-mEtOH, and vortex.
7. Incubate at 60 °C for 30 min at 1200 RPM (in a thermoshaker).
8. Transfer 750 µl to a new tube (at this stage, the sample is divided into two tubes, the original and the new one, both containing ~750 µl). To each tube, add 1 ml of chloroform: isoamyl alcohol (24:1).
9. Mix by inversion (20 times) and centrifuge at 12,500 RPM for 15 min at RT.
10. Transfer the aqueous phase from both tubes into a new 1.5 ml tube, up to 500 µl. If more than 500 µl, create two tubes per sample.
11. Add 1 ml of cold isopropanol (stored at -20 °C) and incubate at -80 °C for 30 min or overnight at -20 °C.
12. Centrifuge at maximum speed for 20 min at 4 °C.
13. Discard the supernatant carefully into a beaker without losing the pellet (if the pellet is loose, remove the supernatant with a micropipette).
14. Add 1 ml of cold 70% EtOH (stored at -20 °C).
15. Centrifuge at 12,000 RPM for 7 min at 4 °C.
16. Repeat steps 13, 14, and 15.
17. Remove the EtOH and air-dry the pellet (it can also be dried on a heating plate at 50–60 °C but monitor to avoid over-drying).
18. Add 35–50 µl of TE buffer and let sit at room temperature for 10 min.
19. Vortex until the pellet dissolves, then add 1 µl of RNase (10 mg/µl) and incubate at 37 °C for 30 min at 500 RPM.
20. Vortex and combine both replicates (if applicable) into a single tube. Label with the sample code and date, then store at 4 °C.

Suspension buffer (pH 8) containing 50 mM EDTA, 120 mM Tris-HCl, 1 M NaCl, 0.5 M sucrose, 2% Triton-X 100.

Extraction buffer containing 20 mM EDTA, 100 mM Tris-HCl, 1.5 M NaCl, 2% CTAB.

**Supplementary figures.**


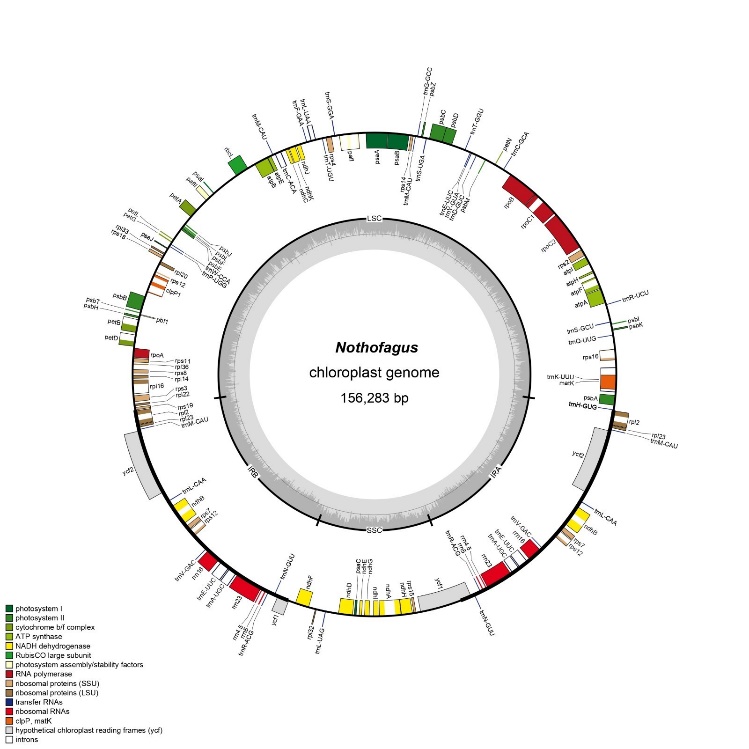

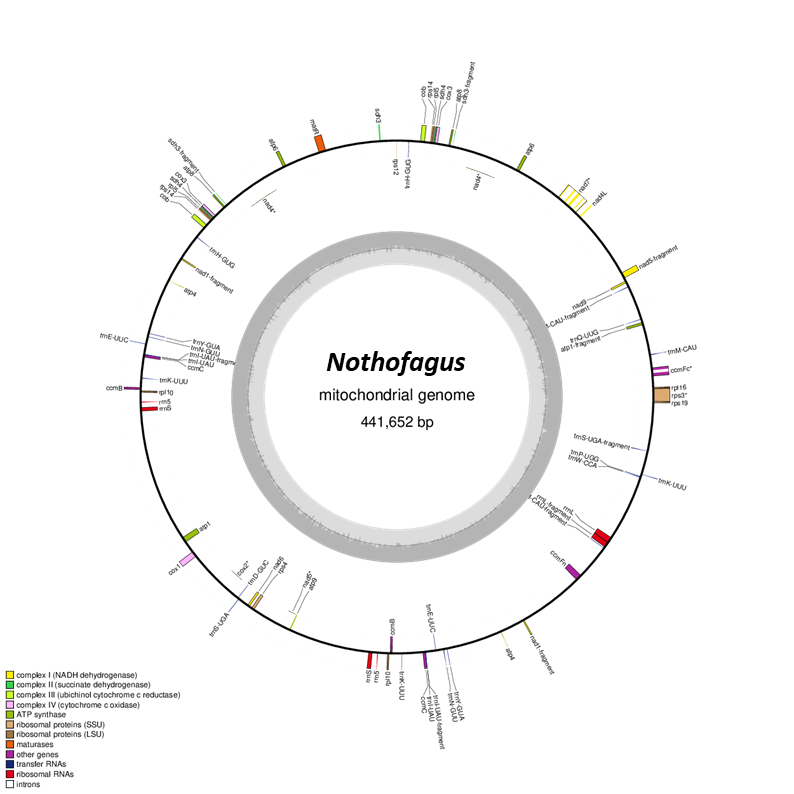


**Figure S1. Representative chloroplast (left) and mitochondrial (right) genome maps of *Nothofagus* spp.** Genes located inside the circle are transcribed clockwise, and genes outside are transcribed counterclockwise. The light gray inner circle corresponds to the AT content and the dark gray to the GC content. Genes belonging to different functional groups are shown in different colors. For the chloroplast genome, the position of the LSC region, SSC region, and two IRs (IRa and IRb) are also indicated. In the figure, the chloroplast genome corresponds to *Nothofagus antarctica* Chillán and the mitochondrial genome belongs to *Nothofagus betuloides* Lago Verde.


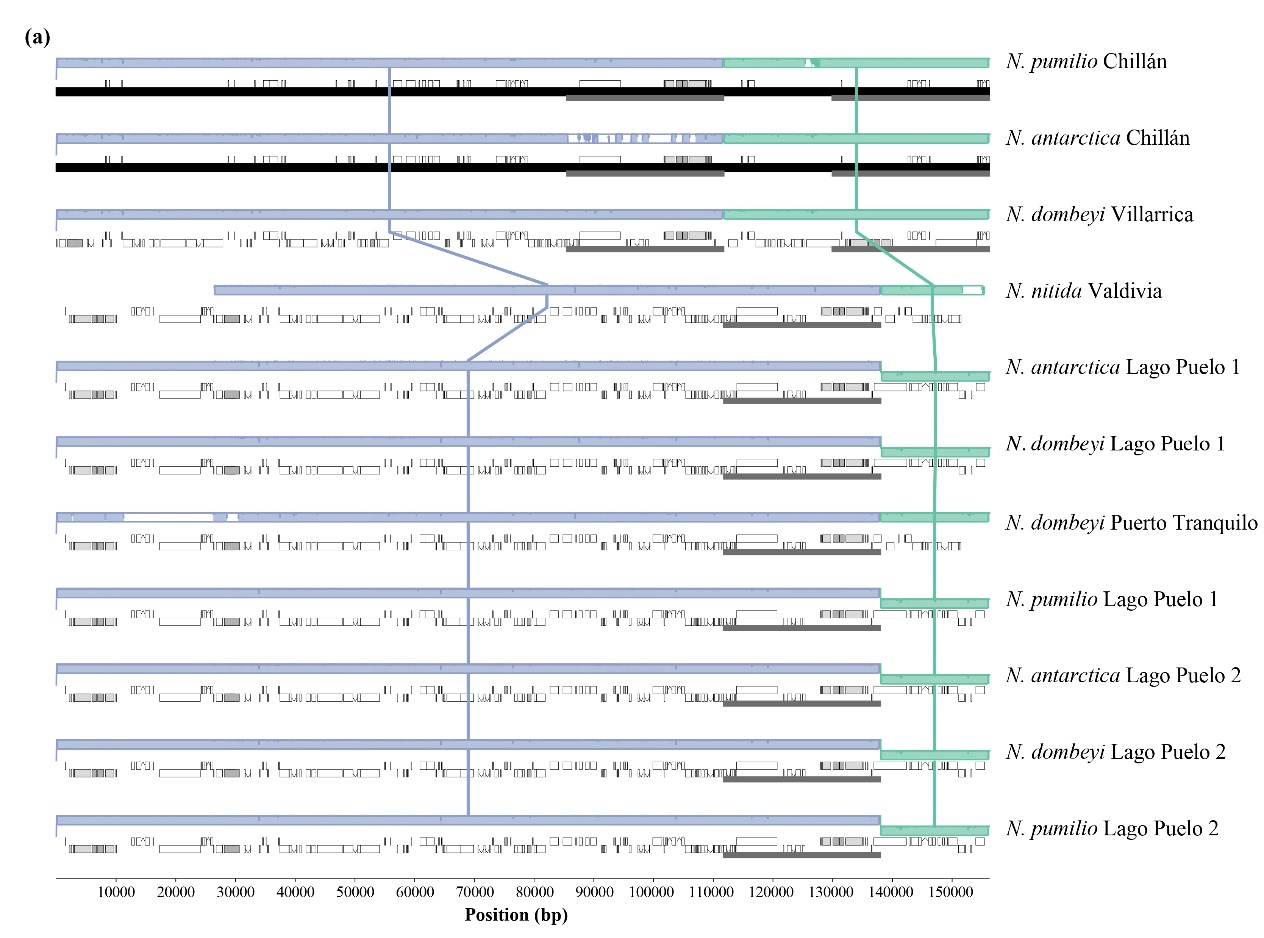

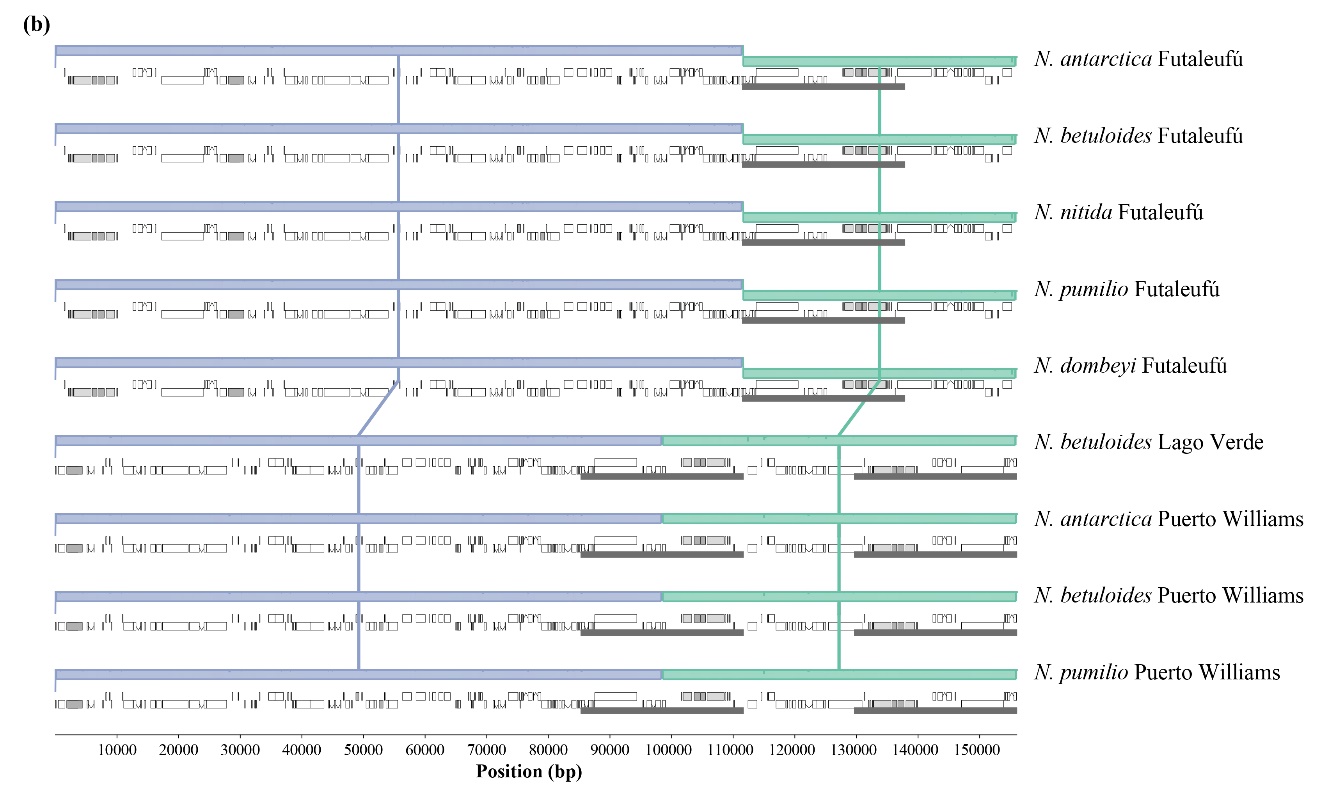


**Figure S2. Comparison of the complete chloroplast genome of all datasets to *Nothofagu*s (samples are described in Table S1).** Two independent Mauve analysis are presented including (a) all samples of the Northern clade and (b) all samples of the Southern clade. More details on sample clustering within the Northern and Southern clade are given in Figure 2b. Local collinear blocks are represented by blocks of the same color connected by vertical lines. Horizontal short gray lines represent IRa and IRb regions respectively.


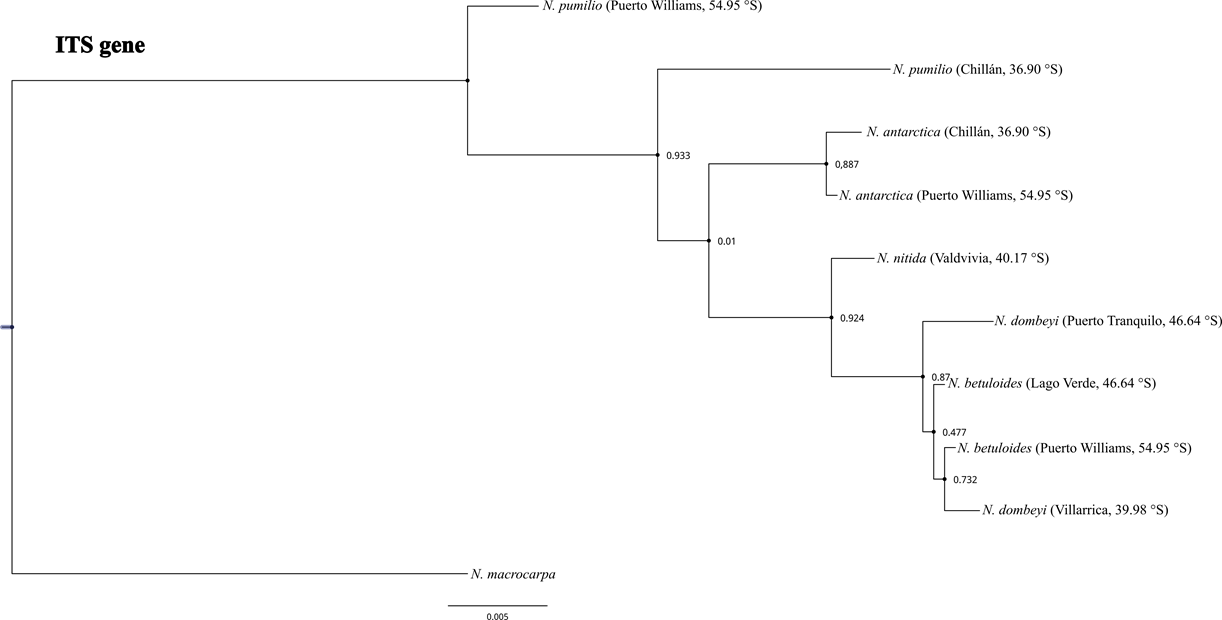


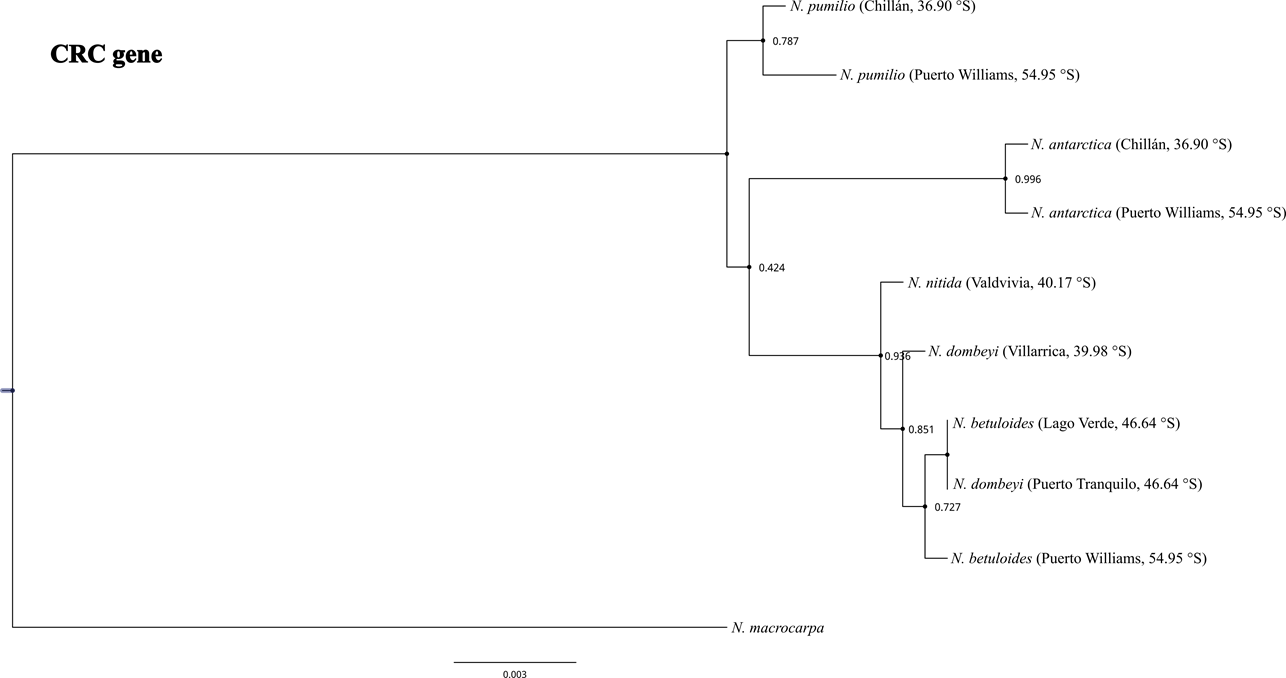


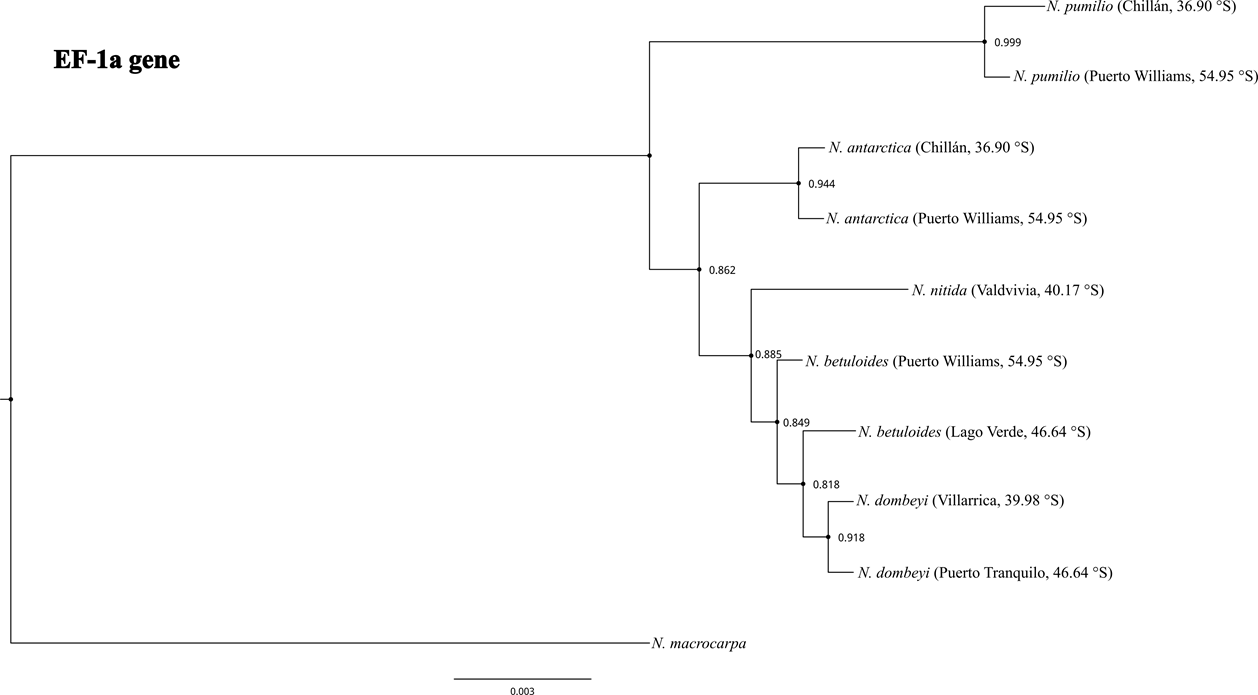


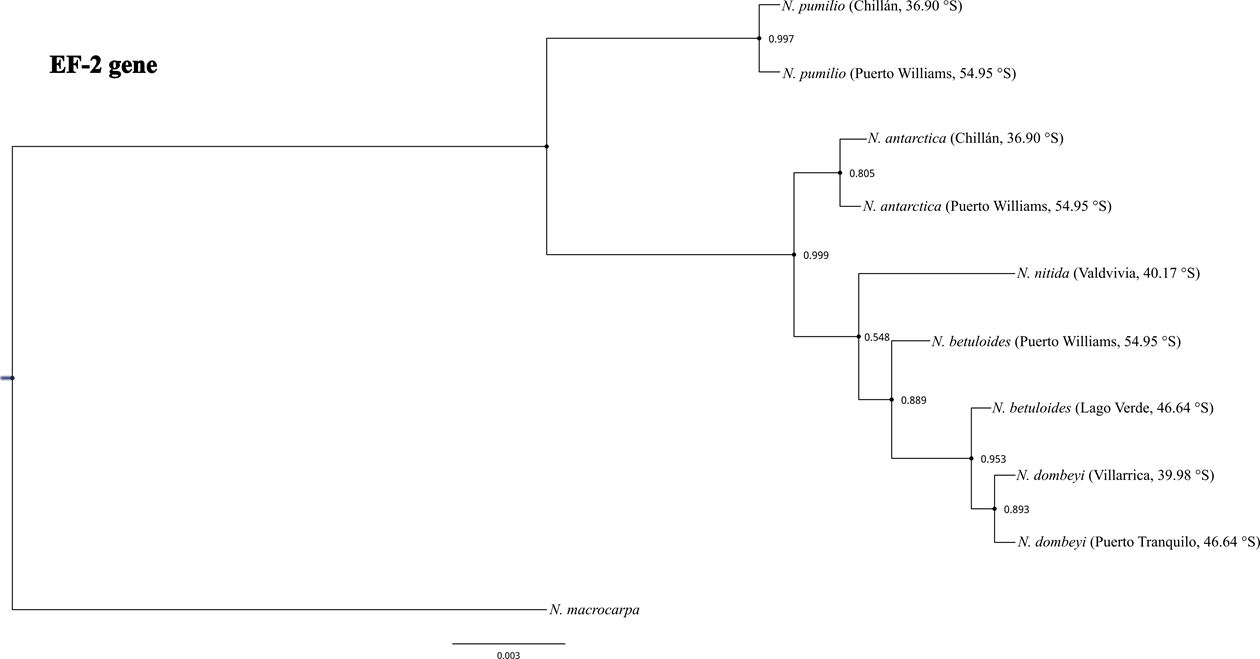


**Figure S3: Phylogenetic tree reconstruction based on nuclear genes.** Maximum Likelihood (ML) tree inferred from ITS, CRC, EF-1a and EF-2 genes, respectively.


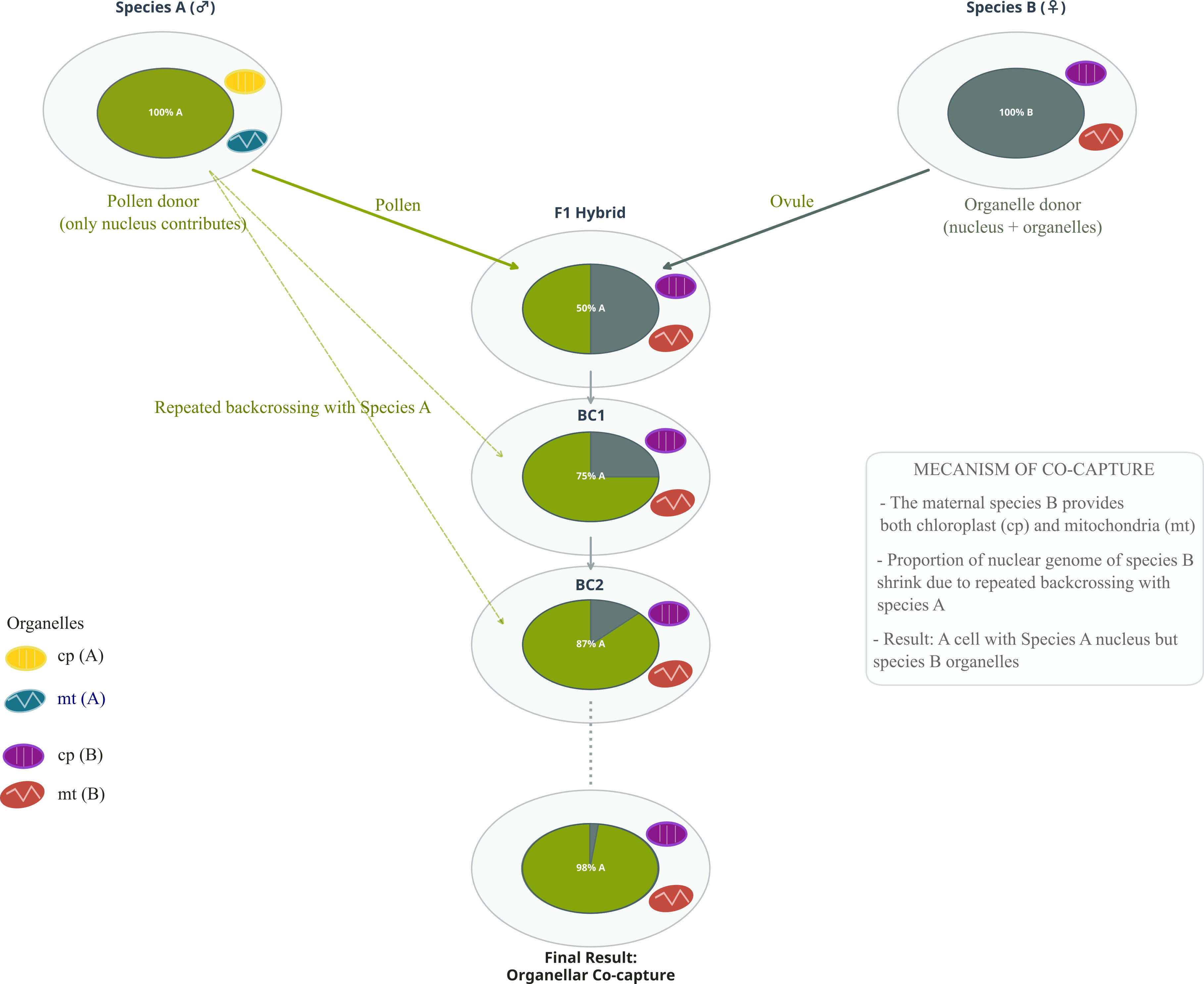


**Figure S4: Schematic representation of organellar co-capture.** Maternal organelles are transferred and captured during hybridization and successive backcrossing events in this schematic representation.

**Supplementary tables.**

**Table S1. Samples coordinates of *Nothofagus spp*. and GenBank accession number.**

| Species | Locality | Latitude | Longitude | GenBank accession number – nuclear genes (ITS, CRC, EF-1a and EF-2) | GenBank accession number – cp genome | GenBank accession number – mt genes | Reference |
| --- | --- | --- | --- | --- | --- | --- | --- |
| *N. antarctica* | Chillán | −36.90 | −71.40 | PX442532, PZ238776- PZ238778 | PX440272 | PX521717- PX521729 | This study |
| *N. antarctica* | Puerto Williams | −54.95 | −67.63 | PX442495, PZ238773- PZ238775 | PX440273 | PX521613- PX521625 | This study |
| *N. antarctica* | Lago Puelo 1 | −41.98 | −71.48 | - | SAMN31781989 | - | Juri et al., 2024 |
| *N. antarctica* | Lago Puelo 2 | −42.21 | −71.73 | - | SAMN31781990 | - | Juri et al., 2024 |
| *N. antarctica* | Futaleufú | −43.00 | −71.52 | - | SAMN31781991 | - | Juri et al., 2024 |
| *N. betuloides* | Lago Verde | −44.26 | −71.82 | PX442431, PZ238755- PZ238757 | PX440274 | PX521626- PX521638 | This study |
| *N. betuloides* | Puerto Williams | −54.95 | −67.63 | PX442419, PZ238752- PZ238754 | PX440275 | PX521639- PX521651 | This study |
| *N. betuloides* | Futaleufú | −43.22 | −71.95 | - | SAMN31781992 | - | Juri et al., 2024 |
| *N. dombeyi* | Villarrica | −39.38 | −71.97 | PX442492, PZ238767- PZ238769 | PX440277 | PX521665- PX521677 | This study |
| *N. dombeyi* | Puerto Tranquilo | −46.64 | −72.68 | PX442493, PZ238770- PZ238772 | PX440276 | PX521652- PX521664 | This study |
| *N. dombeyi* | Lago Puelo 1 | −41.94 | −71.56 | - | SAMN31781993 | - | Juri et al., 2024 |
| *N. dombeyi* | Lago Puelo 2 | −42.10 | −71.72 | - | SAMN31781994 | - | Juri et al., 2024 |
| *N. dombeyi* | Futaleufú | −42.11 | −71.72 | - | SAMN31781995 | - | Juri et al., 2024 |
| *N. nitida* | Valdivia | −40.17 | −73.47 | PX442494, PZ238764- PZ238766 | PX440278 | PX521678- PX521690 | This study |
| *N. nitida* | Futaleufú | −43.01 | −72.50 | - | SAMN31781996 | - | Juri et al., 2024 |
| *N. pumilio* | Chillán | −36.90 | −71.40 | PX442535, PZ238761- PZ238763 | PX440279 | PX521691- PX521703 | This study |
| *N. pumilio* | Puerto Williams | −54.95 | −67.63 | PX442533, PZ238758- PZ238760 | PX440280 | PX521704- PX521716 | This study |
| *N. pumilio* | Lago Puelo 1 | −41.88 | −71.58 | - | SAMN31781997 | - | Juri et al., 2024 |
| *N. pumilio* | Lago Puelo 2 | −42.03 | −71.67 | - | SAMN31781998 | - | Juri et al., 2024 |
| *N. pumilio* | Futaleufú | −42.21 | −71.73 | - | SAMN31781999 | - | Juri et al., 2024 |
| *N. macrocarpa* | Doñihue | −34.16 | −70.97 | PX442348, PZ238779- PZ238781 | PX440271 | PX521600- PX521612 | This study |

**Table S2. Total sequence length and structural characterization of chloroplast genomes of six *Nothofagus* species from this study.** Samples are ordered following the tree presented in Figure 2b.

| Species | LSC  CDS \| tRNA \| rRNA | | | SSC  CDS \| tRNA \| rRNA | | | IRa  CDS \| tRNA \| rRNA | | | IRb  CDS \| tRNA \| rRNA | | | Total length  and coding genes † |
| --- | --- | --- | --- | --- | --- | --- | --- | --- | --- | --- | --- | --- | --- |
| *N. antarctica* Chillán | 85,507 bp | | | 18,136 bp | | | 26,320 bp | | | 26,320 bp | | | 156,283 bp |
|  | 61 | 22 | 0 | 11 | 1 | 0 | 6 | 7 | 4 | 6 | 7 | 4 | 84 |
| *N. pumilio* Chillán | 85,480 bp | | | 18,134 bp | | | 26,320 bp | | | 26,320 bp | | | 156,254 bp |
|  | 61 | 22 | 0 | 11 | 1 | 0 | 6 | 7 | 4 | 6 | 7 | 4 | 84 |
| *N. dombeyi* Villarrica | 85,486 bp | | | 18,140 bp | | | 26,318 bp | | | 26,318 bp | | | 156,262 bp |
|  | 61 | 22 | 0 | 11 | 1 | 0 | 6 | 7 | 4 | 6 | 7 | 4 | 84 |
| *N. nitida*  Valdivia | 85,486 bp | | | 18,131 bp | | | 26,343 bp | | | 26,342 bp | | | 156,302 bp |
|  | 61 | 22 | 0 | 11 | 1 | 0 | 6 | 7 | 4 | 6 | 7 | 4 | 84 |
| *N. dombeyi* Puerto Tranquilo | 85,480 bp | | | 18,144 bp | | | 26,320 bp | | | 26,319 bp | | | 156,263 bp |
|  | 61 | 22 | 0 | 11 | 1 | 0 | 6 | 7 | 4 | 6 | 7 | 4 | 84 |
| *N. betuloides* Lago Verde | 85,371 bp | | | 18,019 bp | | | 26,272 bp | | | 26,272 bp | | | 156,014 bp |
|  | 61 | 22 | 0 | 11 | 1 | 0 | 6 | 7 | 4 | 6 | 7 | 4 | 84 |
| *N. antarctica* Puerto Williams | 85,376 bp | | | 18,105 bp | | | 26,272 bp | | | 26,272 bp | | | 156,025 bp |
|  | 61 | 22 | 0 | 11 | 1 | 0 | 6 | 7 | 4 | 6 | 7 | 4 | 84 |
| *N. betuloides* Puerto Williams | 85,376 bp | | | 18,105 bp | | | 26,272 bp | | | 26,272 bp | | | 156,025 bp |
|  | 61 | 22 | 0 | 11 | 1 | 0 | 6 | 7 | 4 | 6 | 7 | 4 | 84 |
| *N. pumilio*  Puerto Williams | 85,376 bp | | | 18,105 bp | | | 26,272 bp | | | 26,272 bp | | | 156,025 bp |
|  | 61 | 22 | 0 | 11 | 1 | 0 | 6 | 7 | 4 | 6 | 7 | 4 | 84 |
| *N. macrocarpa*  OUTGROUP | 85,213 bp | | | 18,518 bp | | | 26,096 bp | | | 26,096 bp | | | 155,923 bp |
|  | 61 | 22 | 0 | 11 | 1 | 0 | 6 | 7 | 4 | 6 | 7 | 4 | 84 |

†Table does not considered the *rps*12 gene due to trans splicing.

**Table S3. Gene composition of chloroplast genomes of six species of *Nothofagus spp*.**

| Group of genes | Gene name |
| --- | --- |
| Protein coding gene (85) | *acc*D, *atp*A, *atp*B, *atp*E, *atp*F, *atp*H, *atp*I, *ccs*A, *cem*A, *clp*P1, *inf*A*, mat*K, *ndh*A, *ndh*B (×2), *ndh*C, *ndh*D, *ndh*E, *ndh*F, *ndh*G*, ndh*H, *ndh*I, *ndh*J, *ndh*K, *paf*I, *paf*II, *pbf*1, *pet*A, *pet*B, *pet*D, *pet*G, *pet*L, *pet*N, *psa*A, *psa*B, *psa*C, *psa*I, *psa*J, *psb*A, *psb*B, *psb*C, *psb*D, *psb*E*, psb*F*, psb*H*, psb*I*, psb*J*, psb*K, *psb*L, *psb*M*, psb*T, *psb*Z, *rbc*L, *rpl*2 (×2), *rpl*14, *rpl*16, *rpl*20, *rpl*22, *rpl*23 (×2), *rpl*32, *rpl*33, *rpl*36, *rpo*A, *rpo*B, *rpo*C1, *rpo*C2, *rps*2*, rps*3, *rps*4, *rps*7 (×2), *rps*8, *rps*11, *rps*12, *rps*14, *rps*15, *rps*16, *rps*18, *rps*19*, ycf*1 (×2), *ycf*2 (×2) |
| Transfer RNA genes (37) | *trn*A-UGC (×2), *trn*C-GCA, *trn*D-GUC*, trn*E-UUC, *trn*F-GAA, *trn*fM-CAU *trn*G-GCC, *trn*G-UCC, *trn*H-GUG, *trn*I-CAU (×2), *trn*I-GAU (×2)*, trn*K-UUU, *trn*L-CAA (×2), *trn*L-UAA, *trn*L-UAG,  *trn*M-CAU*, trn*N-GUU (×2), *trn*P-UGG, *trn*Q-UUG, *trn*R-ACG (×2), *trn*R-UCU, *trn*S-GCU, *trn*S-GGA, *trn*S-UGA, *trn*T-GGU, *trn*T-UGU, *trn*V-GAC (×2), *trn*V-UAC, *trn*W-CCA, *trn*Y-GUA |
| Ribosomal RNA genes (8) | *rrn*16S (×2), *rrn*23S (×2), *rrn*4.5S (×2), *rrn*5S (×2) |

**Table S4. Gene composition of mitochondrial genomes of two samples of *Nothofagus,* one of *N. dombeyi* (Villarrica) and one of *N. betuloides* (Lago Verde), for which assembled and circularized sequences were obtained.**

| Group of genes | Gene name |
| --- | --- |
| Protein coding gene (32) | *atp*1, *atp*4†, *atp*6, ***atp*8**, *atp*9, ***ccm*B**, ***ccm*C**, ***ccm*Fc**, *ccm*Fn, ***cob***, ***cox*1**, ***cox*2**, ***cox*3**, *mat*R, *nad*1, *nad*2, *nad*4, ***nad*4L**, *nad*5, *nad*6, *nad*7, ***nad*9**, ***rpl*5**, ***rpl*10**, ***rpl*16**, *rps*3, *rps*4, *rps*12, *rps*14, *rps*19, *sdh*3, *sdh*4 |
| Transfer RNA genes (12) | *trn*D-GUC, *trn*E-UUC, *trn*H-GUG, *trn*I-UAU, *trn*K-UUU, *trn*M-CAU, *trn*N-GUU, *trn*P-UGG, *trn*Q-UUG, *trn*S-UGA, *trn*W-CCA, *trn*Y-GUA |
| Ribosomal RNA genes (3) | *rrn*5, *rrn*L, *rrn*S |

Genes shown in bold represent 13 mitochondrial orthologous genes used in mitochondrial phylogeny. †Absent in *N. betuloides* mitogenome.

**Table S5: Summary of read mapping statistics and sequencing depth for 79 chloroplast protein-coding genes across *Nothofagus* species. Values represent the number of mapped reads, with average fold-coverage (X) indicated in parentheses obtained thought raw reads.**

| Gene name | Gene length (bp) | *N. antarctica* Chillán  (12,199X) | *N. pumilio* Chillán (1,174X) | *N. dombeyi* Villarrica (3,463X) | *N. nitida* Valdivia  (13,091X) | *N. dombeyi* Pto. Tranquilo (3,955X) | *N. betuloides* Lago Verde (7,303X) | *N. antarctica*  Pto. Williams (6,044X) | *N. betuloides* Pto. Williams (1,324X) | *N. pumilio*  Pto. Williams (1,316X) | *N. macrocarpa*  OUTGROUP (2,961X) |
| --- | --- | --- | --- | --- | --- | --- | --- | --- | --- | --- | --- |
| *acc*D | 1497 | 113,623 | 11,530 | 34,686 | 114,811 | 40,282 | 74,092 | 52,484 | 12,739 | 12,371 | 26,633 |
|  |  | (10,288X) | (1,050X) | (3,131X) | (10,643X) | (3,676X) | (6,797X) | (4,867X) | (1,151X) | (1,104X) | (2,457X) |
| *atp*A | 1524 | 136,837 | 12,696 | 37,110 | 125,125 | 39,042 | 69,802 | 56,093 | 13,101 | 14,086 | 30,796 |
|  |  | (12,115X) | (1,125X) | (3,219X) | (11,351X) | (3,443X) | (6,261X) | (5,151X) | (1,153X) | (1,230X) | (2,732X) |
| *atp*B | 1497 | 121,487 | 11,483 | 32,239 | 115,500 | 37,479 | 68,977 | 68,657 | 12,378 | 13,780 | 26,103 |
|  |  | (11,006X) | (1,030X) | (2,875X) | (10,493X) | (3,392X) | (6,259X) | (6,436X) | (1,094X) | (1,214X) | (2,338X) |
| *atp*E | 402 | 41,431 | 3,430 | 9,745 | 35,353 | 11,429 | 20,880 | 13,058 | 3,901 | 3,780 | 7,584 |
|  |  | (11,535X) | (1,008X) | (2,715X) | (9,830X) | (3,321X) | (5,975X) | (4,061X) | (1,126X) | (1,065X) | (2,236X) |
| *atp*F | 555 | 57,067 | 4,542 | 14,093 | 45,130 | 14,712 | 30,071 | 18,799 | 5,589 | 4,920 | 13,394 |
|  |  | (11,233X) | (892X) | (2,805X) | (9,243X) | (2,946X) | (6,019X) | (3,564X) | (1,123X) | (968X) | (2,654X) |
| *atp*H | 246 | 22,665 | 2,497 | 6,104 | 21,826 | 6,815 | 13,965 | 11,959 | 2,741 | 2,763 | 6,483 |
|  |  | (9,332X) | (1,028X) | (2,535X) | (9,098X) | (2,890X) | (5,823X) | (5,170X) | (1,114X) | (1,113X) | (2,736X) |
| *atp*I | 774 | 77,526 | 6,972 | 18,445 | 66,549 | 20,754 | 39,370 | 37,148 | 7,448 | 7,434 | 18,127 |
|  |  | (12,706X) | (1,133X) | (3,027X) | (11,313X) | (3,400X) | (6,527X) | (6,070X) | (1,225X) | (1,196X) | (2,967X) |
| *ccs*A | 966 | 91,167 | 9,041 | 23,710 | 90,881 | 26,935 | 52,659 | 57,196 | 9,917 | 8,446 | 19,582 |
|  |  | (11,433X) | (1,133X) | (2,972X) | (11,781X) | (3,398X) | (6,682X) | (7,530X) | (1,243X) | (1,029X) | (2,466X) |
| *cem*A | 690 | 43,781 | 5,600 | 16,678 | 56,840 | 18,815 | 34,975 | 17,655 | 6,188 | 5,916 | 13,287 |
|  |  | (8,173X) | (1,055X) | (3,099X) | (10,872X) | (3,537X) | (6,586X) | (3,403X) | (1,145X) | (1,067X) | (2,432X) |
| *clp*P1 | 591 | 77,213 | 6,174 | 17,209 | 50,957 | 20,037 | 37,241 | 33,244 | 6,526 | 6,917 | 14,280 |
|  |  | (12,824X) | (1,021X) | (2,837X) | (8,363X) | (3,390X) | (6,223X) | (5,728X) | (1,040X) | (1,109X) | (2,364X) |
| *inf*A | 213 | 14,496 | 2,076 | 6,265 | 26,281 | 7,272 | 12,980 | 10,212 | 2,369 | 2,402 | 4,161 |
|  |  | (6,712X) | (1,007X) | (2,984X) | (12,188X) | (3,566X) | (6,335X) | (5,473X) | (1,159X) | (1,158X) | (1,927X) |
| *mat*K | 1524 | 94,167 | 11,649 | 34,113 | 125,768 | 39,661 | 77,197 | 56,247 | 13,125 | 12,789 | 29,686 |
|  |  | (8,026X) | (986X) | (2,911X) | (10,923X) | (3,383X) | (6,644X) | (4,632X) | (1,117X) | (1,054X) | (2,432X) |
| *ndh*A | 1092 | 97,033 | 7,235 | 27,797 | 113,348 | 30,202 | 59,245 | 42,761 | 10,797 | 10,135 | 22,439 |
|  |  | (9,974X) | (793X) | (2,958X) | (12,081X) | (3,220X) | (6,340X) | (4,651X) | (1,142X) | (1,059X) | (2,391X) |
| *ndh*B | 1533 | 196,907 | 27,489 | 73,673 | 297,467 | 84,530 | 151,118 | 161,955 | 28,420 | 29,888 | 65,824 |
|  |  | (16,901X) | (2,294X) | (6,160X) | (25,070X) | (7,155X) | (12,789X) | (13,857X) | (2,390X) | (2,455X) | (5,492X) |
| *ndh*C | 363 | 31,838 | 2,761 | 8,410 | 32,709 | 9,441 | 18,966 | 10,646 | 3,110 | 3,354 | 7,006 |
|  |  | (10,647X) | (908X) | (2,766X) | (10,685X) | (3,140X) | (6,121X) | (3,355X) | (988X) | (1,047X) | (2,257X) |
| *ndh*D | 1536 | 139,160 | 13,252 | 36,434 | 123,996 | 36,766 | 76,025 | 35,270 | 13,986 | 13,086 | 27,538 |
|  |  | (12,033X) | (1,138X) | (3,102X) | (11,044X) | (3,244X) | (6,631X) | (3,030X) | (1,188X) | (1,090X) | (2,367X) |
| *ndh*E | 306 | 43,512 | 2,940 | 9,264 | 29,343 | 9,248 | 19,986 | 12,808 | 3,424 | 3,086 | 7,522 |
|  |  | (16,740X) | (1,106X) | (3,471X) | (10,940X) | (3,491X) | (7,263X) | (4,821X) | (1,201X) | (1,089X) | (2,702X) |
| *ndh*F | 1458 | 130,362 | 10,655 | 31,725 | 116,197 | 30,098 | 70,257 | 39,561 | 12,485 | 11,682 | 28,016 |
|  |  | (12,039X) | (952X) | (2,821X) | (10,276X) | (3,117X) | (6,271X) | (3,605X) | (1,107X) | (1,000X) | (2,460X) |
| *ndh*G | 531 | 56,586 | 5,327 | 13,951 | 51,290 | 15,375 | 30,270 | 15,228 | 5,421 | 5,333 | 11,283 |
|  |  | (13,348X) | (1,244X) | (3,210X) | (11,978X) | (3,611X) | (7,038X) | (3,654X) | (1,235X) | (1,191X) | (2,596X) |
| *ndh*H | 1182 | 105,813 | 3,737 | 27,164 | 108,331 | 28,918 | 58,511 | 32,421 | 10,963 | 10,470 | 22,319 |
|  |  | (12,027X) | (457X) | (2,997X) | (12,332X) | (3,216X) | (6,589X) | (3,551X) | (1,211X) | (1,137X) | (2,470X) |
| *ndh*I | 510 | 69,210 | 7,025 | 21,150 | 50,490 | 22,859 | 33,534 | 22,496 | 8,328 | 6,571 | 16,890 |
|  |  | (15,118X) | (1,438X) | (4,232X) | (11,475X) | (4,383X) | (7,570X) | (5,196X) | (1,691X) | (1,362X) | (3,170X) |
| *ndh*J | 477 | 27,331 | 4,319 | 10,890 | 42,845 | 13,154 | 25,086 | 19,737 | 4,389 | 4,695 | 9,959 |
|  |  | (6,307X) | (1,090X) | (2,641X) | (10,788X) | (3,304X) | (6,259X) | (4,864X) | (1,087X) | (1,150X) | (2,367X) |
| *ndh*K | 678 | 45,346 | 5,114 | 14,582 | 57,091 | 16,580 | 33,285 | 27,997 | 5,893 | 6,104 | 11,985 |
|  |  | (8,660X) | (1,000X) | (2,786X) | (10,940X) | (3,207X) | (6,224X) | (5,397X) | (1,091X) | (1,121X) | (2,293X) |
| *paf*I | 507 | 49,551 | 4,700 | 14,125 | 37,581 | 16,797 | 30,448 | 33,514 | 5,392 | 5,488 | 12,776 |
|  |  | (9,707X) | (899X) | (2,677X) | (7,162X) | (3,225X) | (5,880X) | (6,551X) | (1,034X) | (1,037X) | (2,415X) |
| *paf*II | 555 | 50,369 | 4,982 | 14,326 | 50,631 | 15,121 | 29,213 | 17,994 | 5,300 | 5,245 | 11,281 |
|  |  | (10,242X) | (1,073X) | (3,032X) | (11,313X) | (3,233X) | (6,311X) | (3,676X) | (1,146X) | (1,130X) | (2,404X) |
| *pbf*1 | 132 | 16,493 | 1,421 | 4,054 | 15,907 | 4,679 | 8,185 | 6,781 | 1,643 | 1,696 | 3,012 |
|  |  | (10,292X) | (867X) | (2,502X) | (9,567X) | (2,899X) | (5,099X) | (3,916X) | (1,022X) | (1,005X) | (1,845X) |
| *pet*A | 963 | 81,709 | 8,143 | 22,846 | 74,948 | 25,963 | 48,277 | 32,289 | 8,452 | 8,695 | 17,390 |
|  |  | (11,066X) | (1,099X) | (3,068X) | (10,537X) | (3,520X) | (6,640X) | (4,562X) | (1,127X) | (1,143X) | (2,383X) |
| *pet*B | 648 | 56,434 | 4,750 | 13,860 | 46,697 | 15,633 | 27,981 | 21,107 | 5,089 | 5,572 | 11,046 |
|  |  | (11,567X) | (961X) | (2,763X) | (9,527X) | (3,103X) | (5,623X) | (4,270X) | (1,011X) | (1,089X) | (2,224X) |
| *pet*D | 483 | 54,447 | 4,412 | 12,747 | 39,349 | 15,026 | 26,094 | 19,726 | 4,854 | 4,995 | 10,902 |
|  |  | (12,992X) | (1,067X) | (3,076X) | (10,074X) | (3,675X) | (6,314X) | (4,823X) | (1,195X) | (1,199X) | (2,635X) |
| *pet*G | 114 | 15,496 | 1,535 | 4,755 | 16,356 | 5,513 | 10,059 | 7,329 | 1,763 | 1,719 | 4,252 |
|  |  | (10,330X) | (1,048X) | (3,146X) | (11,151X) | (3,660X) | (6,724X) | (4,882X) | (1,198X) | (1,112X) | (2,753X) |
| *pet*L | 96 | 18,665 | 1,357 | 4,331 | 13,049 | 4,709 | 8,491 | 5,296 | 1,715 | 1,366 | 3,293 |
|  |  | (14,463X) | (1,033X) | (3,279X) | (9,614X) | (3,553X) | (6,397X) | (4,084X) | (1,315X) | (1,016X) | (2,526X) |
| *pet*N | 90 | 8,160 | 1,208 | 3,274 | 11,678 | 3,937 | 7,135 | 4,388 | 1,188 | 1,224 | 3,218 |
|  |  | (6,124X) | (961X) | (2,539X) | (8,869X) | (3,037X) | (5,550X) | (3,476X) | (942X) | (933X) | (2,506X) |
| *psa*A | 2253 | 188,761 | 17,241 | 48,154 | 175,638 | 54,919 | 99,612 | 95,423 | 18,839 | 20,715 | 40,008 |
|  |  | (11,842X) | (1,063X) | (2,933X) | (10,926X) | (3,385X) | (6,185X) | (5,977X) | (1,149X) | (1,250X) | (2,412X) |
| *psa*B | 2205 | 165,230 | 16,166 | 47,219 | 180,170 | 56,756 | 98,147 | 113,258 | 17,880 | 19,938 | 39,344 |
|  |  | (10,312X) | (1,012X) | (2,942X) | (11,475X) | (3,581X) | (6,204X) | (7,376X) | (1,111X) | (1,215X) | (2,450X) |
| *psa*C | 246 | 25,913 | 2,692 | 7,201 | 21,536 | 8,252 | 15,838 | 11,698 | 2,686 | 2,640 | 5,607 |
|  |  | (9,780X) | (1,136X) | (2,981X) | (9,478X) | (3,516X) | (6,801X) | (5,028X) | (1,121X) | (1,092X) | (2,382X) |
| *psa*I | 114 | 17,616 | 1,291 | 3,485 | 6,561 | 4,249 | 7,432 | 4,481 | 1,344 | 1,329 | 2,587 |
|  |  | (12,834X) | (945X) | (2,530X) | (4,544X) | (3,214X) | (5,440X) | (3,397X) | (979X) | (,930X) | (1,901X) |
| *psa*J | 135 | 19,381 | 1,602 | 4,873 | 13,003 | 5,959 | 10,088 | 6,725 | 1,924 | 1,671 | 3,812 |
|  |  | (11,773X) | (948X) | (2,969X) | (8,213X) | (3,706X) | (6,398X) | (4,277X) | (1,196X) | (1,040X) | (2,283X) |
| *psb*A | 1062 | 94,258 | 8,030 | 24,274 | 89,700 | 27,678 | 53,271 | 63,969 | 9,279 | 9,500 | 20,218 |
|  |  | (11,468X) | (984X) | (2,965X) | (11,534X) | (3,441X) | (6,607X) | (8,022X) | (1,156X) | (1,148X) | (2,495X) |
| *psb*B | 1527 | 105,968 | 11,781 | 31,791 | 124,569 | 37,498 | 66,937 | 66,096 | 12,082 | 14,152 | 27,925 |
|  |  | (9,579X) | (1,053X) | (2,831X) | (11,253X) | (3,370X) | (6,070X) | (6,146X) | (1,082X) | (1,251X) | (2,463X) |
| *psb*C | 1416 | 85,487 | 9,926 | 27,933 | 103,050 | 33,144 | 58,766 | 63,288 | 11,257 | 12,893 | 27,033 |
|  |  | (8,468X) | (967X) | (2,721X) | (10,198X) | (3,257X) | (5,853X) | (6,457X) | (1,090X) | (1,229X) | (2,630X) |
| *psb*D | 1062 | 86,932 | 7,252 | 21,469 | 81,320 | 24,179 | 43,375 | 56,340 | 8,625 | 9,100 | 20,546 |
|  |  | (11,403X) | (930X) | (2,792X) | (10,592X) | (3,168X) | (5,723X) | (7,691X) | (1,120X) | (1,153X) | (2,686X) |
| *psb*E | 252 | 29,052 | 2,935 | 7,158 | 28,421 | 8,699 | 15,686 | 14,140 | 2,799 | 2,880 | 5,938 |
|  |  | (10,970X) | (1,158X) | (2,818X) | (11,189X) | (3,419X) | (6,264X) | (6,115X) | (1,102X) | (1,116X) | (2,382X) |
| *psb*F | 120 | 20,552 | 1,500 | 4,475 | 16,603 | 5,092 | 9,197 | 8,854 | 1,662 | 1,496 | 3,693 |
|  |  | (12,148X) | (804X) | (2,455X) | (8,542X) | (2,845X) | (5,146X) | (4,563X) | (904X) | (813X) | (2,015X) |
| *psb*H | 222 | 26,219 | 2,130 | 6,229 | 21,729 | 6,997 | 12,827 | 9,982 | 2,457 | 2,393 | 4,559 |
|  |  | (10,796X) | (900X) | (2,569X) | (8,294X) | (3,006X) | (5,493X) | (4,889X) | (990X) | (943X) | (1,944X) |
| *psb*I | 111 | 8,230 | 1,079 | 3,014 | 5,817 | 3,498 | 6,889 | 7,623 | 1,177 | ,884 | 2,426 |
|  |  | (4,478X) | (657X) | (1,765X) | (3,041X) | (2,114X) | (4,140X) | (5,054X) | (,688X) | (511X) | (1,477X) |
| *psb*J | 123 | 14,510 | 1,723 | 5,059 | 17,407 | 5,969 | 10,335 | 7,558 | 1,935 | 1,887 | 3,756 |
|  |  | (9,704X) | (1,129X) | (3,386X) | (11,794X) | (3,941X) | (6,819X) | (5,233X) | (1,274X) | (1,234X) | (2,472X) |
| *psb*K | 186 | 27,689 | 2,081 | 5,852 | 18,618 | 6,504 | 12,864 | 10,782 | 2,157 | 2,081 | 5,154 |
|  |  | (14,125X) | (1,055X) | (3,005X) | (9,503X) | (3,363X) | (6,661X) | (5,196X) | (1,079X) | (1,052X) | (2,506X) |
| *psb*L | 117 | 24,915 | 1,733 | 5,639 | 19,120 | 6,242 | 10,708 | 10,978 | 1,917 | 1,964 | 4,135 |
|  |  | (13,600X) | (973X) | (3,152X) | (12,232X) | (3,411X) | (5,949X) | (4,574X) | (1,068X) | (1,091X) | (2,195X) |
| *psb*M | 105 | 18,973 | 1,593 | 4,096 | 12,281 | 4,507 | 10,123 | 8,754 | 1,772 | 1,739 | 4,048 |
|  |  | (11,921X) | (970X) | (2,748X) | (8,932X) | (3,012X) | (6,446X) | (5,252X) | (1,071X) | (1,040X) | (2,609X) |
| *psb*T | 108 | 10,968 | 1,076 | 3,360 | 17,304 | 3,598 | 6,756 | 4,168 | 1,392 | 1,311 | 2,637 |
|  |  | (7,593X) | (771X) | (2,403X) | (11,679X) | (2,545X) | (4,812X) | (3,031X) | (1,028X) | (912X) | (1,717X) |
| *psb*Z | 189 | 17,467 | 1,920 | 5,652 | 21,283 | 7,040 | 11,914 | 8,905 | 2,158 | 2,055 | 4,860 |
|  |  | (9,095X) | (947X) | (2,857X) | (10,965X) | (3,608X) | (6,096X) | (4,373X) | (1,131X) | (1,042X) | (2,431X) |
| *rbc*L | 1428 | 97,246 | 11,561 | 31,540 | 116,162 | 35,473 | 66,246 | 77,884 | 12,108 | 13,553 | 25,691 |
|  |  | (9,261X) | (1,120X) | (3,029X) | (11,267X) | (3,431X) | (6,428X) | (7,715X) | (1,160X) | (1,277X) | (2,461X) |
| *rpl*2 | 825 | 144,028 | 16,801 | 43,118 | 147,200 | 51,969 | 91,024 | 133,140 | 15,775 | 18,373 | 38,799 |
|  |  | (18,129X) | (2,254X) | (5,754X) | (20,772X) | (6,993X) | (12,476X) | (17,382X) | (2,143X) | (2,420X) | (5,331X) |
| *rpl*14 | 369 | 31,291 | 2,888 | 8,419 | 30,035 | 9,506 | 17,833 | 12,493 | 3,234 | 3,314 | 7,758 |
|  |  | (9,167X) | (857X) | (2,492X) | (8,755X) | (2,907X) | (5,398X) | (4,473X) | (961X) | (979X) | (2,301X) |
| *rpl*16 | 408 | 51,632 | 3,427 | 10,064 | 28,662 | 10,546 | 20,096 | 15,318 | 3,762 | 3,637 | 8,320 |
|  |  | (15,274X) | (996X) | (2,893X) | (8,413X) | (3,078X) | (5,870X) | (4,679X) | (1,098X) | (1,058X) | (2,393X) |
| *rpl*20 | 357 | 38,809 | 2,921 | 9,001 | 31,008 | 9,759 | 19,572 | 15,030 | 3,528 | 3,345 | 6,793 |
|  |  | (12,664X) | (944X) | (2,848X) | (9,917X) | (3,171X) | (6,360X) | (5,251X) | (1,140X) | (1,071X) | (2,200X) |
| *rpl*22 | 168 | 36,458 | 2,512 | 7,383 | 19,854 | 8,328 | 11,788 | 13,167 | 2,096 | 2,069 | 5,740 |
|  |  | (17,397X) | (1,177X) | (3,417X) | (9,116X) | (3,922X) | (6,526X) | (8,597X) | (1,136X) | (1,120X) | (3,043X) |
| *rpl*23 | 282 | 88,092 | 5,857 | 17,159 | 64,119 | 19,753 | 35,093 | 43,308 | 6,115 | 6,525 | 14,927 |
|  |  | (32,785X) | (2,103X) | (6,252X) | (23,717X) | (7,171X) | (12,918X) | (16,042X) | (2,219X) | (2,272X) | (5,418X) |
| *rpl*32 | 156 | 23,475 | 1,770 | 5,682 | 12,145 | 6,046 | 12,004 | 4,728 | 2,403 | 1,738 | 4,512 |
|  |  | (12,946X) | (961X) | (3,149X) | (7,279X) | (3,377X) | (6,698X) | (2,656X) | (1,285X) | (903X) | (2,393X) |
| *rpl*33 | 201 | 19,057 | 2,084 | 5,994 | 24,447 | 6,983 | 12,621 | 7,692 | 2,464 | 2,319 | 4,402 |
|  |  | (9,501X) | (1,043X) | (2,933X) | (11,980X) | (3,501X) | (6,277X) | (4,046X) | (1,159X) | (1,146X) | (2,219X) |
| *rpl*36 | 114 | 9,422 | 1,401 | 4,650 | 16,151 | 4,903 | 9,303 | 8,372 | 1,858 | 1,337 | 3,541 |
|  |  | (6,637X) | (948X) | (3,160X) | (10,779X) | (3,422X) | (6,428X) | (7,535X) | (1,245X) | (897X) | (2,539X) |
| *rpo*A | 996 | 71,420 | 8,175 | 23,793 | 77,517 | 25,596 | 51,001 | 35,208 | 8,838 | 8,938 | 21,087 |
|  |  | (8,362X) | (1,015X) | (2,931X) | (9,956X) | (3,191X) | (6,761X) | (4,771X) | (1,123X) | (1,124X) | (2,662X) |
| *rpo*B | 3219 | 216,167 | 24,809 | 65,379 | 248,559 | 75,301 | 137,353 | 98,957 | 26,217 | 27,072 | 65,203 |
|  |  | (9,053X) | (1,041X) | (2,728X) | (10,587X) | (3,162X) | (5,798X) | (4,258X) | (1,093X) | (1,110X) | (2,723X) |
| *rpo*C1 | 2049 | 177,542 | 16,155 | 46,063 | 165,870 | 49,681 | 91,310 | 81,385 | 17,140 | 17,646 | 42,866 |
|  |  | (11,514X) | (1,047X) | (2,962X) | (10,584X) | (3,205X) | (5,932X) | (5,332X) | (1,103X) | (1,125X) | (2,776X) |
| *rpo*C2 | 4167 | 337,767 | 32,429 | 89,053 | 328,856 | 100,748 | 184,945 | 156,452 | 34,896 | 35,180 | 82,759 |
|  |  | (11,462X) | (1,094X) | (2,990X) | (11,252X) | (3,417X) | (6,317X) | (5,429X) | (1,173X) | (1,159X) | (2,820X) |
| *rps*2 | 711 | 50,543 | 5,285 | 14,802 | 51,194 | 16,911 | 31,496 | 25,003 | 5,974 | 6,069 | 15,624 |
|  |  | (8,795X) | (917X) | (2,548X) | (9,074X) | (2,960X) | (5,518X) | (4,551X) | (1,022X) | (1,037X) | (2,666X) |
| *rps*3 | 657 | 55,534 | 5,646 | 16,417 | 46,200 | 18,636 | 33,881 | 31,182 | 5,957 | 5,753 | 15,216 |
|  |  | (10,613X) | (1,110X) | (3,247X) | (9,609X) | (3,713X) | (6,803X) | (6,301X) | (1,182X) | (1,124X) | (2,959X) |
| *rps*4 | 606 | 49,457 | 4,727 | 13,137 | 47,685 | 15,151 | 28,506 | 24,033 | 5,499 | 5,073 | 12,118 |
|  |  | (10,111X) | (985X) | (2,699X) | (9,906X) | (3,149X) | (5,953X) | (4,894X) | (1,138X) | (1,031X) | (2,466X) |
| *rps*7 | 468 | 90,974 | 8,425 | 24,006 | 93,379 | 26,131 | 47,402 | 29,332 | 8,832 | 9,220 | 21,284 |
|  |  | (23,850X) | (2,190X) | (6,271X) | (24,143X) | (6,823X) | (12,407X) | (7,566X) | (2,288X) | (2,358X) | (5,383X) |
| *rps*8 | 405 | 41,964 | 3,711 | 11,272 | 36,402 | 12,567 | 22,923 | 12,300 | 4,451 | 4,143 | 9,323 |
|  |  | (11,930X) | (1,041X) | (3,194X) | (10,667X) | (3,582X) | (6,485X) | (3,380X) | (1,242X) | (1,139X) | (2,615X) |
| *rps*11 | 417 | 44,959 | 4,171 | 11,297 | 36,512 | 12,781 | 23,252 | 20,002 | 4,260 | 4,455 | 9,619 |
|  |  | (11,613X) | (1,125X) | (3,013X) | (10,216X) | (3,463X) | (6,285X) | (5,863X) | (1,137X) | (1,177X) | (2,579X) |
| *rps*12 | 369 | 45,226 | 6,751 | 16,391 | 57,197 | 15,137 | 36,055 | 50,178 | 6,906 | 8,280 | 15,596 |
|  |  | (11,068X) | (1,730X) | (4,161X) | (22,591X) | (5,930X) | (9,350X) | (12,938X) | (1,741X) | (2,084X) | (4,000X) |
| *rps*14 | 303 | 35,586 | 2,697 | 8,253 | 27,836 | 8,868 | 15,681 | 15,410 | 3,170 | 3,105 | 6,467 |
|  |  | (13,453X) | (969X) | (2,986X) | (10,123X) | (3,201X) | (5,774X) | (5,146X) | (1,143X) | (1,087X) | (2,369X) |
| *rps*15 | 273 | 32,300 | 134 | 8,853 | 29,273 | 9,203 | 18,406 | 9,580 | 3,256 | 2,897 | 6,497 |
|  |  | (12,049X) | (54X) | (3,381X) | (11,196X) | (3,606X) | (7,193X) | (4,243X) | (1,233X) | (1,043X) | (2,622X) |
| *rps*16 | 255 | 26,503 | 2,290 | 6,327 | 24,876 | 8,273 | 19,436 | 15,696 | 3,700 | 3,161 | 5,575 |
|  |  | (7,021X) | (731X) | (1,798X) | (8,127X) | (2,487X) | (5,929X) | (4,885X) | (1,139X) | (969X) | (1,770X) |
| *rps*18 | 306 | 30,143 | 2,768 | 7,929 | 27,722 | 8,470 | 16,765 | 8,919 | 2,964 | 2,847 | 6,403 |
|  |  | (11,116X) | (994X) | (2,912X) | (10,028X) | (3,168X) | (6,276X) | (3,673X) | (1,066X) | (1,026X) | (2,281X) |
| *rps*19 | 279 | 32,413 | 2,825 | 7,822 | 23,070 | 9,184 | 17,253 | 15,998 | 2,870 | 2,920 | 7,068 |
|  |  | (12,790X) | (1,096X) | (3,162X) | (8,564X) | (3,588X) | (6,799X) | (6,172X) | (1,100X) | (1,119X) | (2,948X) |
| *ycf*1 | 5460 | 551,732 | 36,513 | 152,351 | 223,820 | 62,456 | 312,879 | 172,440 | 56,979 | 52,048 | 120,774 |
|  |  | (13,025X) | (877X) | (3,582X) | (24,225X) | (6,493X) | (7,534X) | (4,121X) | (1,346X) | (1,202X) | (2,836X) |
| *ycf*2 | 6822 | 945,124 | 103,659 | 307,430 | 1,198,948 | 329,260 | 610,154 | 458,414 | 114,906 | 115,245 | 255,615 |
|  |  | (18,940X) | (2,081X) | (6,139X) | (24,421X) | (6,620X) | (12,391X) | (9,355X) | (2,275X) | (2,260X) | (5,113X) |

**Table S6: Summary of read mapping statistics and sequencing depth for 13 mitochondrial protein-coding genes across *Nothofagus* species. Values represent the number of mapped reads, with average fold-coverage (X) indicated in parentheses obtained thought raw reads.**

| Gene name | Gene length (bp) | *N. antarctica* Chillán  (1,037X) | *N. pumilio* Chillán (184X) | *N. dombeyi* Villarrica (595X) | *N. nitida* Valdivia (1,553X) | *N. dombeyi* Pto. Tranquilo (595X) | *N. betuloides* Lago Verde (393X) | *N. antarctica*  Pto. Williams (551X) | *N. betuloides* Pto. Williams (190X) | *N. pumilio*  Pto. Williams (189X) | *N. macrocarpa*  OUTGROUP (362X) |
| --- | --- | --- | --- | --- | --- | --- | --- | --- | --- | --- | --- |
| *atp*8 | 480 | 5,629 (1,453X) | 1,191 (288X) | 3,847 (940X) | 10,581 (2,685X) | 3,657 (940X) | 2,613 (633X) | 3,621 (884X) | 480  (300X) | 1,283 (297X) | 1,939 (419X) |
| *ccm*B | 621 | 6,051 (1,232X) | 979  (195X) | 3,143 (643X) | 8,312 (1,699X) | 2,501 (643X) | 1,654 (341X) | 2,449 (506X) | 621  (189X) | 1,034 (199X) | 2,032 (403X) |
| *ccm*C | 753 | 5,817 (984X) | 1,028 (170X) | 3,369 (556X) | 9,536 (1,580X) | 2,588 (556X) | 2,021 (335X) | 2,569 (437X) | 753  (161X) | 1,196 (199X) | 2,005 (329X) |
| *ccm*Fc | 1,347 | 9,477 (824X) | 1,742 (148X) | 5,290 (468X) | 12,301 (1,091X) | 4,147 (468X) | 3,433 (307X) | 4,730 (433X) | 1,347 (173X) | 1,800 (145X) | 3,496 (315X) |
| *cob* | 1,182 | 9,359 (1009x) | 1,678 (183X) | 4,657 (506X) | 11,860 (1,329X) | 4,677 (506X) | 3,535 (389X) | 5,831 (668X) | 1,182 (188X) | 1,848 (196X) | 3,397 (373X) |
| *cox*1 | 1,584 | 15,444 (1,296X) | 2,277 (186X) | 7,779 (627X) | 19,675 (1,615X) | 6,824 (627X) | 4,758 (380X) | 6,447 (535X) | 1,584 (181X) | 2,013 (155X) | 4,828 (380X) |
| *cox*2 | 774 | 7,919 (1,229X) | 1,182 (162X) | 3,918 (581X) | 10,229 (1,561X) | 3,300 (581X) | 2,801 (414X) | 4,671 (702X) | 774  (186X) | 1,199 (165X) | 2,813 (397X) |
| *cox*3 | 798 | 7,511 (1,192X) | 1,644 (260X) | 5,193 (833X) | 15,158 (2,396X) | 5,046 (833X) | 3,700 (596X) | 4,781 (791X) | 798  (255X) | 1,807 (285X) | 2,277 (345X) |
| *nad*4L | 303 | 2,780 (815X) | 528  (176X) | 1,884 (614X) | 5,022 (1,602X) | 1,509 (614X) | 1,281 (436X) | 1,154 (374X) | 303  (165X) | 520  (167X) | 1,063 (356X) |
| *nad*9 | 573 | 3,575 (742X) | 727  (153X) | 2,666 (556X) | 6,905 (1,405X) | 2,118 (556X) | 1,703 (359X) | 2,153 (470X) | 573  (157X) | 771  (154X) | 1,541 (321X) |
| *rpl*5 | 558 | 3,458 (745X) | 653  (145X) | 2,107 (459X) | 5,445 (1,212X) | 1,861 (459X) | 1,707 (371X) | 1,779 (382X) | 558  (142X) | 783  (158X) | 1,622 (359X) |
| *rpl*10 | 489 | 3,880 (930X) | 797  (198X) | 2,494 (599X) | 5,540 (1,336X) | 2,189 (599X) | 1,459 (353X) | 2,487 (614X) | 489  (205X) | 822  (205X) | 1,459 (354X) |
| *rpl*16 | 435 | 3,547 (936X) | 540  (144X) | 1,773 (479X) | 4,275 (1,176X) | 1,465 (479X) | 1,189 (311X) | 1,292 (338X) | 435  (128X) | 625  (167X) | 1,322 (343X) |

**Table S7. Variation in chloroplast gene length among *Nothofagus spp*.**

| Gene | Length retrieved in most samples sequenced (pb) | Variation (pb) |
| --- | --- | --- |
| *acc*D | 1,497 | 1,494 in *Nothofagus macrocarpa* |
| *inf*A | 213 | 204 in *Nothofagus macrocarpa* |
| *ndh*F | 1,458 | 1,257 in *Nothofagus macrocarpa* |
| *ndh*K | 678 | 675 in *Nothofagus macrocarpa* |
| *psb*T | 108 | 114 in *Nothofagus macrocarpa* |
| *rps*3 | 657 | 672 in *Nothofagus macrocarpa* |
| *rps*16 | 255 | 270 in *Nothofagus macrocarpa* |
| *rps*18 | 306 | 321 in *Nothofagus macrocarpa* |
| *rpl*22 | 228 in samples part of the Northern clade † | 168 in samples part of the Southern clade †  180 in *Nothofagus macrocarpa* |
| *rpo*A | 981 in samples part of the Northern clade † | 996 in samples part of the Southern clade †  981 in *Nothofagus macrocarpa* |
| *ycf*1 large copy | 5,475 in samples part of the Northern clade † | 5,460 in samples part of the Southern clade †  5,460 in *Nothofagus macrocarpa* |
| *ycf*2 short and large copy | 6,816 in samples part of the Northern clade † | 6,822 in samples part of the Southern clade †  6,828 in *Nothofagus macrocarpa* |
| *ccs*A | 966 | 972 in *Nothofagus nitida* (Valdivia) † |
| *nhd*I | 522 in all samples part of the Northern clade † and 510 in samples part of the Southern clade †. | 516 in *Nothofagus dombeyi* (Villarrica)  534 in *Nothofagus dombeyi* (Puerto Tranquilo)  513 in *Nothofagus macrocarpa* |

† More details on samples clustering in the Northern and Southern clade are given in Figure 2b.
